# Plant Signaling Hormones and Transcription Factors: Key Regulators of Plant Responses to Growth, Development, and Stress

**DOI:** 10.3390/plants14071070

**Published:** 2025-03-31

**Authors:** Ayomi S. Thilakarathne, Fei Liu, Zhongwei Zou

**Affiliations:** 1 Department of Biology, Wilfrid Laurier University, Waterloo, ON N2L 3C5, Canada; thil4220@mylaurier.ca; 2 School of Life Sciences, Henan University, Kaifeng 475001, China; liuhuijuedui@163.com

**Keywords:** biotic and abiotic stress, phytohormones, plant signaling, plant–pathogen interaction, transcription factors

## Abstract

Plants constantly encounter a wide range of biotic and abiotic stresses that adversely affect their growth, development, and productivity. Phytohormones such as abscisic acid, jasmonic acid, salicylic acid, and ethylene serve as crucial regulators, integrating internal and external signals to mediate stress responses while also coordinating key developmental processes, including seed germination, root and shoot growth, flowering, and senescence. Transcription factors (TFs) such as WRKY, NAC, MYB, and AP2/ERF play complementary roles by orchestrating complex transcriptional reprogramming, modulating stress-responsive genes, and facilitating physiological adaptations. Recent advances have deepened our understanding of hormonal networks and transcription factor families, revealing their intricate crosstalk in shaping plant resilience and development. Additionally, the synthesis, transport, and signaling of these molecules, along with their interactions with stress-responsive pathways, have emerged as critical areas of study. The integration of cutting-edge biotechnological tools, such as CRISPR-mediated gene editing and omics approaches, provides new opportunities to fine-tune these regulatory networks for enhanced crop resilience. By leveraging insights into transcriptional regulation and hormone signaling, these advancements provide a foundation for developing stress-tolerant, high-yielding crop varieties tailored to the challenges of climate change.

## 1. Introduction

Plants have acquired sophisticated sensing mechanisms and cognitive abilities to cope with diverse stresses [1]. Stresses can be divided into two major classes, abiotic stresses and biotic stresses. Abiotic stresses are caused by non-living factors such as salinity, drought, cold, heat, heavy metals, and UV light exposure, while biotic stresses are caused when plants get attacked by pathogenic or parasitic organisms, such as viruses, bacteria, fungi, or insects [2]. Such stresses negatively impact plant growth and development, ultimately reducing agricultural output. This has become a serious global concern, causing a more than 50% yield loss in major crops annually [3,4]. However, plants have evolved a series of defense mechanisms to manage these recurring environmental stresses. These include signal perception, transduction, and activation of stress-responsive genes, which subsequently trigger physiological and metabolic responses [5,6].

Various signal transduction pathways work together in plants to respond to abiotic and biotic stimuli. These responses involve complex interactions among plant hormones, reactive oxygen species (ROS), receptors, calcium ions, kinases, phosphatases, regulatory proteins, and numerous small molecules [7,8]. Plant signaling hormones, also known as phytohormones, are small natural compounds that act as chemical messengers to coordinate growth, development, and defense processes in plants [9]. They integrate internal developmental cues with external environmental signals to elicit appropriate responses. By modulating the growth rate and development of tissue as well as influencing organ lifespan, plant hormones play a crucial role in the adaptation of plants to ever-changing environments [10]. Under stress conditions, phytohormones mitigate stresses by activating defense mechanisms. They regulate physiological and biochemical processes to enhance plant tolerance to environmental challenges [11].

Plants have developed advanced immune systems to defend against pathogens, relying on two main types of innate immunity: PAMP-triggered immunity (PTI) and effector-triggered immunity (ETI) [12,13]. PTI serves as the first line of defense against microbial attacks, while ETI is activated through interactions between plant R proteins and pathogen effector proteins [14,15]. The extensive reprogramming of gene expression during a specific immune response clearly depends on the coordinated activity of various transcription factors (TFs), functioning in a temporal- and spatial-specific manner [16,17].

Understanding the mechanisms by which plants develop and their response to various biotic and abiotic stresses is fundamental for applying plant biotechnology to ensure sustainable agriculture. Plant stress tolerance can be enhanced through innovative strategies, including foliar treatments, genetic engineering, and other biotechnological tools [18,19,20]. These approaches are increasingly critical in the face of climate change, which amplifies the frequency and intensity of environmental stresses. The key to this advancement is unraveling the endogenous processes and molecular mechanisms involved in plant stress responses.

Plant signaling hormones and TFs play central roles in these processes, acting as regulators of stress tolerance by modulating gene expression and activating defense pathways. This review delves into the biosynthesis, signaling pathways, and multifaceted roles in mitigating stress impacts of phytohormones, as well as their complex interplay with TFs, examining their classification, organization, and regulatory activities during stress conditions.

## 2. Plant Signaling Hormones

Plants grow in constantly changing natural environments and often face conditions that are not conducive to their growth or survival [21]. During the life cycle of plants, they are exposed to various living and non-living stressors that can disrupt normal physiological functions, and since plants are sessile organisms, they cannot move to avoid these stresses. Therefore, they have developed advanced mechanisms to withstand, alleviate, or recover by modifying their growth and physiological processes to cope with these challenging conditions [22,23,24,25]. Over the past two decades, research has shown that plant hormones play crucial roles in detecting and transmitting various environmental signals and initiating defense responses.

Plant signaling hormones are a group of small organic signaling molecules that interact at various levels to regulate growth, development, and responses to environmental conditions at very low concentrations [26]. Currently, nine major classes of natural plant hormones have been identified as essential in numerous reactions to environmental signals [27]. These hormones include auxin, cytokinin, gibberellin, abscisic acid, ethylene, brassinosteroid, jasmonic acid, salicylic acid, and strigolactone [25,27]. The balance of these hormones, known as hormone homeostasis, is controlled by their synthesis, metabolism, transport, perception, and signal transduction, which governs their activity within the plant. Hormone perception can occur either near or far from where they are produced. Consequently, hormones can be actively transported to their target sites, ensuring proper distribution and triggering specific responses [28,29,30].

### 2.1. Auxin

Auxin is the first discovered phytohormone, integral to almost every plant growth and development phase, shaping the entire plant body and regulating processes such as apical dominance, root initiation, and tropisms [31,32]. It plays a critical role in regulating root and shoot architecture, maintaining meristems, and facilitating processes like lateral and adventitious root formation, leaf morphogenesis, flowering, and senescence [33,34]. The distribution of auxin within plant tissues is primarily driven by polar auxin transport (PAT), which establishes gradients that influence cell division, elongation and differentiation, the steering of organ patterning, and crucial developmental events [35,36]. In addition, auxin operates through complex signaling pathways that adjust plant growth and development in response to environmental changes and stresses, including metal and metalloid toxicity [37].

The ability of auxin to integrate environmental signals into developmental growth responses is critical for adapting plants to changing environments. The role of auxin extends to facilitating cellular responses, such as cell elongation, expansion, and differentiation, allowing plants to adjust their growth patterns according to external stimuli. Through its comprehensive influence on plant physiology, auxin ensures that plants can modulate their development dynamically, optimizing survival and growth in diverse environmental conditions [38].

Auxin biosynthesis has been a subject of study for more than 70 years. Although the entire process is not yet completely understood, significant advances have been made in the past decade to unravel the molecular mechanisms involved in producing indole-3-acetic acid (IAA), the primary auxin in plants [39,40,41]. The IAA is synthesized through both tryptophan-dependent and tryptophan-independent pathways [42,43]. However, the tryptophan-dependent TAA/YUC (Tryptophan Aminotransferase of *Arabidopsis*/YUCCA) pathway is the most well characterized, involving two sequential steps [44,45]. The TAA family of aminotransferases first converts tryptophan into indole-3-pyruvate (IPyA), followed by the YUC family of flavin-containing monooxygenases catalyzing the oxidative decarboxylation of IPyA to produce IAA. This pathway is essential for major developmental processes, ensuring the proper integration of environmental signals into the plant growth responses, especially under stress conditions [46]. The tryptophan-independent (Trp-independent) pathway is a proposed method for synthesizing IAA. The Trp-independent pathway was originally hypothesized based on data from Trp auxotrophs, which have mutations in the genes that encode the α- or β-subunits of Trp synthase [47]. The Trp-independent pathway is less well understood than the Trp-dependent pathway, which is the well characterized pathway that uses tryptophan as a precursor for IAA. However, recent studies have proved that the Trp-independent pathway is important for early embryogenesis in plants [48].

**Figure 1:**
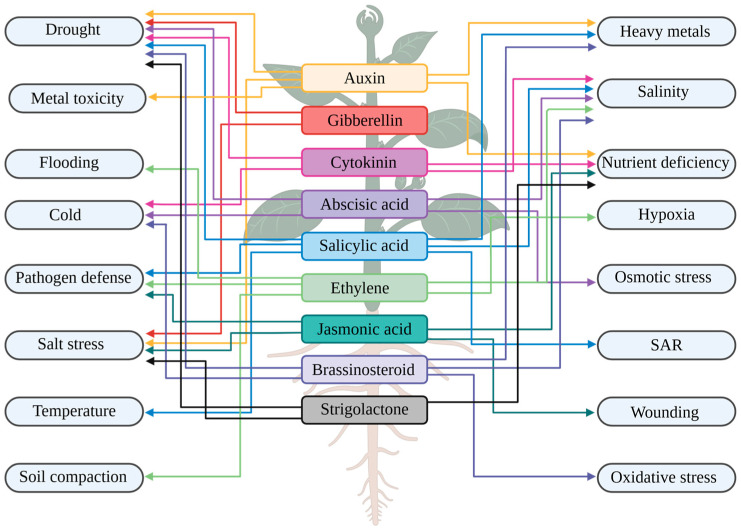
A summary of phytohormones’ participation in stress conditions (created with BioRender).

The AUX1 gene promotes root hair elongation in response to phosphate limitation in both *Arabidopsis thaliana* and rice (*Oryza sativa*), highlighting its role in adapting to nutrient stress (Figure 1) [49,50]. In canola (*Brassica napus*), overexpression of the *BnaA.TTG2.a.1* (*WRKY44*) gene increases sensitivity to salt stress, leading to reduced levels of IAA by downregulating the expression of *TRYPTOPHAN BIOSYNTHESIS 5* (TRP5) and *YUCCA2* (YUC2) genes [51]. Similarly, in *A. thaliana*, overproduction of the multifunctional cytochrome P450 enzyme results in elevated auxin levels, while the double knockout of *CYP79B2* and *CYP79B3* leads to decreased IAA levels and growth defects associated with auxin deficiency [52]. In rice, overexpression of *OsYUCCA1* increases IAA levels and results in auxin overproduction phenotypes. In contrast, suppression of *OsYUCCA1* leads to abnormal growth, mirroring the characteristics of auxin-insensitive rice plants [52]. These findings underscore the critical roles of auxin biosynthesis and regulation in plant stress responses and development.

The integral role of auxin in plant growth, development, and environmental adaptation highlights its potential for applications in improving crop resilience and productivity. By understanding the molecular pathways governing auxin biosynthesis and signaling, we can develop targeted strategies to enhance stress tolerance and optimize growth patterns under adverse conditions.

### 2.2. Gibberellic Acid

Gibberellin/gibberellic acid (GA) is a diverse group of tetracyclic diterpenoid carboxylic acids, with some members acting as growth-promoting hormones in plants [53]. They play crucial roles in a wide range of biological processes, including seed germination, determining plant height, fertility, and fruit ripening [54,55]. Although GAs are often associated with regulating plant stature and seed dormancy, recent research suggests this view may be too simplistic. Along with other phytohormones, GAs affect many plant traits directly or indirectly. They were pivotal in the Green Revolution of the 20th century, and many improved plant varieties with desirable agronomic traits, such as dwarf phenotypes and those with increased biomass, have been linked to GA activity and signaling [56,57]. The potential of GAs remains largely untapped, as they promise to drive a new Green Revolution by enhancing yield and improving nitrogen-use efficiency [58].

GAs are known to positively regulate cell expansion and division, and plants deficient in GAs often experience a reduction in the length of the meristematic zone length and slow development, and remain small [59,60]. In contrast, overexpression of GA biosynthetic pathways or signaling can increase plant size [61,62]. GAs are vital for plant adaptation because external conditions influence their metabolism, and their functions span the entire plant life cycle. They are widely recognized for promoting growth through cell expansion and division and are involved in various developmental processes such as floral transition, male fertility, photomorphogenesis, and fruit setting [63,64,65,66]. Gibberellins are present in many forms in all vascular plants; however, only a few, such as GA_1_, GA_3_, GA_4_, and GA_7_, are bioactive. The rest are non-bioactive precursors or catabolites that support these critical growth processes throughout the life cycle of plants [67].

The biosynthesis of active gibberellins follows a series of enzymatic reactions involving multiple genes expressed in various cells, tissues, and plant developmental stages. GAs are synthesized in vascular plants from the precursor trans-geranylgeranyl diphosphate (GGPP), which is converted to ent-kaurene by the enzymes ent-copalyl diphosphate synthase (CPS) and ent-kaurene synthase (KS) [68]. Subsequently, ent-kaurene is transformed into the intermediate GA12 through the actions of ent-kaurene oxidase (KO) and ent-kaurenoic acid oxidase (KAO) [69,70]. GA_12_ is then further processed into bioactive GAs by the enzymes GA 20-oxidase (GA20ox) and GA 3-oxidase (GA3ox) [71,72]. The activity of these enzymes is crucial, as they regulate the levels of bioactive GAs in plant tissues, with GA20ox and GA3ox acting as rate-limiting steps that increase the pool of active GAs from intermediate or inactive forms. Conversely, active GAs can be deactivated by GA 2-oxidase (GA2ox) enzymes, which convert them into inactive forms [73].

The transport of GAs within the plant includes long-distance and short-distance movement, with GA_12_, a mobile precursor, capable of moving from root to shoot [74]. The perception of GAs is mediated by the nuclear receptor GIBBERELLIN-INSENSITIVE DWARF1 (GID1). Upon GA binding, GID1 facilitates the ubiquitination and degradation of DELLA proteins, which are repressors of GA signaling. DELLA proteins belong to the GRAS family and are key regulators that block GA signaling. The degradation of DELLA proteins upon GA–GID1 interaction allows for GA signaling to proceed, thus regulating growth and development [75]. The continuous balance between GA perception and DELLA degradation facilitates the plant genetic response to GAs by playing a crucial role in numerous developmental processes, such as seed germination, plant height regulation, and fruit ripening, and these have significant agricultural impacts [76,77].

The NPF proteins were the first identified transporters of gibberellins, with NPF3 promoting GA influx in the elongating endodermal cells of *A. thaliana* roots. NPF3 expression is regulated by nitrogen, GA, and abscisic acid, with repression by the former two and induction by the latter [78,79]. Other NPF transporters, such as NPF2.5, NPF4.1, and NPF4.6, have been shown to facilitate GA uptake in *Xenopus* oocytes, while NPF4.1 also plays a role in promoting seed germination in plants [80,81]. The nonspecific transport activity of NPF proteins overlaps with the SWEET family, including GA transporters such as SWEET13 and SWEET14 in *A. thaliana* and SWEET3a in rice [82]. In addition to GA transport, GA biosynthesis is tightly regulated, as evidenced by the downregulation of the *AtGA3ox1* gene (GA4) in *A. thaliana* in response to GA activity. GA-deficient mutants, which often exhibit stunted growth and delayed flowering, further emphasize the importance of GA in plant development [83]. In *Brassica* mutants, similar to nonvernalized winter annuals, the failure to bolt and flower could be attributed to reduced GA levels [84,85].

GAs are crucial for regulating key developmental processes and play a vital role in environmental adaptation. Advances in understanding GA biosynthesis, signaling, and transport offer opportunities to manipulate these pathways to enhance crop productivity and stress resilience. By enhancing the ability of GAs to modulate growth and improve nitrogen-use efficiency, we can develop sustainable agricultural strategies, potentially driving a new Green Revolution to meet global food demands.

### 2.3. Cytokinin

Cytokinins (CK) are vital plant hormones involved in various developmental and physiological processes, such as maintaining shoot and cambial meristem activities, promoting cell division and differentiation, and root modulation [86]. In addition to enhancing cell division and overall plant growth, CKs play a crucial role in delaying plant senescence by preventing the breakdown of chlorophyll, nucleic acids, proteins, and other essential substances. They also help redistribute vital hormones, amino acids, inorganic salts, and other compounds throughout the plant [87]. CKs are mobile molecules that exist in two primary active forms: trans-zeatin (tZ) and N6-(D2-isopentenyl) adenine (iP). Trans-zeatin is synthesized in the roots and moves acropetally to the shoot through the xylem, while N6-(D2-isopentenyl) adenine (iP) is produced in the shoot and travels basipetally toward the root via the phloem [88]. This dynamic distribution and regulation of CKs facilitate their involvement in numerous developmental and physiological processes by ensuring proper growth and adaptation to environmental changes.

CKs exist in plants in at least 32 different forms and are distributed and transported among various plant organs, where they influence numerous biological processes through their diverse chemical properties and distinct metabolic and signaling pathways [89,90]. CK homeostasis is intricately regulated by the actions of two critical enzyme families: isopentenyltransferase (IPT) and cytokinin oxidase/dehydrogenase (CKX) [91]. The central genes currently known to be involved in the cytokinin biosynthesis pathway encode the ISOPENTENYL TRANSFERASE (IPT) and LONELY GUY (LOG) enzymes [92]. IPT enzymes catalyze the first and rate-limiting step of CK biosynthesis, transferring an isopentenyl group from dimethylallyl diphosphate to an adenine nucleotide (ATP, ADP, or AMP) to produce N6-prenylated adenine derivatives. This process results in the formation of CK, with isopentenyl adenine (iP) and trans-zeatin being the most prevalent. The LOG enzymes then remove a ribose 5′-monophosphate group. This crucial step allows CK to bind to their receptors and initiate signal transduction within the cell. Like the IPT family, the LOG gene family comprises nine members expressed in various organs with distinct and overlapping expression domains, often found in vascular tissues [93].

CKX genes are expressed at deficient concentrations across different organs, with specific patterns ensuring precise regulation of CK concentration. CKX enzymes are responsible for CK degradation and cause significant maintenance of CK levels within a cell [90]. CKX proteins are located in various cellular compartments, including the vacuole, cytoplasm, and apoplast, the space between the cell wall and plasma membrane, which are essential for developing various organs, as evidenced by the phenotypes of plants with mutations in these genes [93].

Several *A. thaliana* type-A response regulator genes, including *ARR3*, *ARR6*, *ARR7*, *ARR8*, *ARR9*, *ARR15*, and *ARR16*, were initially identified by their sequence homology to bacterial response regulators [94,95]. These genes were later characterized as CK-induced with varying response kinetics and found to act as negative regulators of CK signaling [96]. In rice, the *OsABCG18* gene, similar to *ABCG14* in *A. thaliana*, regulates the long-distance transport of CK from the roots [97]. Additionally, *OsPUP1* and *OsPUP7* import CK into the endoplasmic reticulum. At the same time, *OsPUP4* facilitates CK transport from the apoplast into the cytosol, collectively controlling grain size and development [98,99]. In *B. napus*, stable transgenic plants expressing AtMYB32xs::IPT exhibit delayed leaf senescence under both controlled and field conditions, further highlighting the role of CK in plant development and stress responses [100].

Understanding the biosynthetic and signaling pathways of CKs, alongside their intricate regulation by IPT and CKX enzymes, provides valuable insights for optimizing their use in agriculture. By leveraging CK-mediated responses, such as delayed senescence and enhanced stress tolerance, we can develop strategies to improve crop yield and resilience in challenging environmental conditions.

### 2.4. Abscisic Acid

Abscisic acid (ABA) is a critical phytohormone that regulates numerous plant processes, including seed dormancy, germination, root development, and growth. It is a small sesquiterpene molecule pivotal in stress responses, particularly in drought tolerance and stomatal closure [101,102]. This hormone is involved in various other abiotic stress responses, such as to salinity, flooding, heat, and cold, and also in many phenological and developmental processes [103,104,105,106], including vegetative growth, flowering, fruit development and ripening, lipid and storage protein synthesis, and leaf senescence [102]. Additionally, ABA plays a role in modulating leaf hydraulic conductance and mesophyll conductance, and it promotes cuticular wax formation, leading to a thicker and less permeable cuticle [107]. The concentration of ABA can lead to varied effects on shoot and root growth depending on the plant species. Notably, elevated levels of ABA inhibit shoot growth during drought conditions while promoting root elongation and inhibiting lateral root formation to enhance water uptake efficiency [108,109].

ABA biosynthesis is a complex process involving multiple enzymes encoded by various genes. It was initially believed to be primarily produced in the roots and transported to the shoots to mediate these responses. However, recent studies have shown that ABA is also synthesized in the shoot, mainly in the vasculature, and can regulate distinct responses throughout the plant, such as stomatal aperture and other aspects of plant–water relations [110]. The ABA biosynthesis pathway begins with the hydroxylation of β-carotene to zeaxanthin in the chloroplast. It is followed by its conversion to violaxanthin and subsequently to neoxanthin through the xanthophyll cycle. Neoxanthin is then isomerized from its 9-*trans* form to the 9-*cis* form before undergoing oxidative cleavage by 9-*cis*-epoxycarotenoid dioxygenase (NCED) to form xanthoxin, which occurs in the chloroplasts and is the first non-reversible and rate-limiting step in ABA biosynthesis. Xanthoxin is converted to ABA in subsequent steps outside the chloroplast [111]. The contribution of specific *NCED* genes varies during different stages of plant development. For example, *NCED2* and *3* show high expression levels in roots and leaves, whichever are less active during seed development [112]. Conversely, *NCED5*, *6*, and *9* are upregulated during later stages of seed maturation, playing a crucial role in ABA accumulation in mature seeds. In *A. thaliana*, this pathway is primarily regulated by the activity of the NCED gene family, which includes *AtNCED2*, *3*, *5*, *6*, and *9* [112].

In *A. thaliana*, members of the ATP Binding Cassette (ABC) family, such as ABCG25 and ABCG40, have been identified as critical transporters of ABA. ABCG25 functions as an ABA exporter from the vasculature. At the same time, ABCG40 imports ABA into guard cells, indicating an active transport mechanism that moves ABA from the vasculature to guard cells [113,114]. Other ABA transporters include DTX50 from the MATE family and AIT1-4 (also known as NPF4.6, 4.5, 4.1, 4.2). The activity of AIT2-4 has been demonstrated in yeast; however, it has not yet been confirmed in plants [115,116,117]. In Barrel clover (*Medicago truncatula*), MtABCG20 is another ABA exporter found in roots and germinating seeds [118]. In *B. napus*, a novel VP14 homolog, *BnNCED3*, was isolated and shown to be stress-responsive in leaves, with its expression increasing during leaf senescence in tandem with ABA accumulation. NCED homologs, crucial for ABA biosynthesis by cleaving C40-cis epoxycarotenoids to produce the ABA precursor xanthoxin, have also been identified in various dicot and monocot plants, including avocados (*Persea americana*), cowpeas (*Vigna unguiculata*), beans (*Phaseolus vulgaris*), rice, and wheat (*Triticum aestivum*) [119,120,121,122].

ABA is a versatile phytohormone essential for regulating growth, development, and abiotic stress responses, particularly drought tolerance and stomatal control. By advancing our understanding of ABA biosynthesis, signaling, and transport mechanisms, we can develop strategies to enhance crop resilience to drought and other environmental stresses. These insights pave the way for innovations in agricultural practices, including breeding or engineering crops with optimized ABA pathways to sustain productivity under changing climate conditions.

### 2.5. Ethylene

Ethylene (ET), a simple molecule composed of two carbon and four hydrogen atoms, is one of the earliest discovered and most important plant hormones [123]. It is widely used as a plant growth regulator in agricultural practices. ET plays a crucial role in various growth and development processes, including organ development, seed germination, fertilization, cell elongation, senescence, abscission, fruit ripening, and seed dispersal [124]. Ethylene also plays a vital role in responding to biotic and abiotic stresses such as pathogenic attacks, flooding, high salt levels, and soil compaction [125,126]. This volatile hormone can be found in a wide range of plants, including angiosperms, gymnosperms, ferns, and mosses, and it diffuses quickly from production sites after biosynthesis without being modified or metabolized [127,128].

The ethylene biosynthesis pathway in plants involves a series of well-regulated enzymatic steps. It begins with the precursor methionine, which is converted into S-adenosyl-L-methionine (SAM) by the enzyme SAM synthetase (SAMS), a multigene family protein. SAM is a precursor in ethylene biosynthesis and plays a role in other biochemical pathways, such as polyamine synthesis. Then, the SAM is converted into 1-aminocyclopropane-1-carboxylic acid (ACC) by ACC synthase (ACS), with the byproduct 5′-methylthioadenosine (MTA) feeding back into a “methionine cycle” for efficient methionine utilization. Finally, ACC is oxidized by ACC oxidase (ACO) to produce ethylene and byproducts CO_2_ and cyanide [129,130]. These three key enzymes, SAMS, ACS, and ACO, are tightly regulated during ethylene synthesis [131]. Ethylene biosynthesis is initiated by endogenous developmental signals, such as growth, ripening, and senescence, as well as by external biotic and abiotic factors [132,133]. The ET signaling pathway involves receptors (ETR1, ERS1, ETR2, EIN4, ERS2), the protein kinase CTR1, and EIN2, which then signal to TFs like EIN3, EIL1, and EIL2, ultimately leading to ethylene-mediated responses in the plant [134].

In pea (*Pisum sativum*), the hormones gibberellins, auxins, and ethylene play crucial roles in seed and fruit development. For pea fruit pericarps, high levels of endogenous auxins, including IAA and 4-chloro-IAA, are linked to increased expression of the gibberellin biosynthesis gene *GA3ox1* and are also associated with reduced ethylene production [135,136,137]. Also, several Yang cycle genes are upregulated in plant organs with high ethylene production. For instance, *SlMTN*, *SlMTK*, and *SlARD1-2* are induced in tomatoes (*Solanum lycopersicum*) during climacteric fruit ripening, while *OsMTN* and *OsARD1* show increased expression in rice under submergence [138,139,140]. A rice line with enhanced expression of OsARD also exhibited higher ethylene production, which contributed to improved tolerance to submergence [141]. While ethylene production is higher in canola seeds than in their silique walls, the roles of ethylene in canola seed growth and silique wall development remain unclear [142]. However, studies have suggested that ethylene evolution in desiccating siliques and seeds of mustard and canola may contribute to seed degreening [143]. Moreover, elevated ethylene production can be observed during specific pathogen infections in *A. thaliana*. The *Pseudomonas syringae* effector HopAF1 suppresses ethylene production by interfering with MTN activity, ultimately increasing disease susceptibility [144].

By understanding the intricate biosynthetic and signaling pathways of ethylene, including its interaction with other phytohormones like gibberellins and auxins, researchers can explore its potential to optimize crop productivity and stress resilience. Applications of ethylene management in agriculture promise to enhance seed quality, extend shelf life, and improve stress adaptation in diverse plant species.

### 2.6. Brassinosteroid

Brassinosteroids (BRs) are a class of essential steroidal phytohormones involved in a wide range of physiological and biochemical processes in plants, including cell division, elongation, morphogenesis, reproduction, and senescence [145]. BRs also play a crucial role in helping plants respond to environmental stresses such as heavy metals, drought, salinity, and extreme temperatures [146]. Brassinolide (BL) is the most active BR, which was first isolated from *B. napus* pollen in 1979, and its chemical structure was determined through crystal diffraction analysis [147]. Since then, extensive research has established BRs as the sixth most crucial group of endogenous plant hormones, alongside auxin, GA, CK, ABA, and ET. Over 70 naturally occurring compounds similar to BL have been identified, and these compounds are found in lower and higher plants, particularly angiosperms, and are collectively referred to as BRs [148]. They are present in all plant organs, including roots, stems, leaves, flowers, seeds, and grains, and exist in various forms, such as conjugated with fatty acids and sugars or glucosides and sulfates [149,150,151]. Additionally, they enhance plant tolerance to abiotic stress by modulating the activity of enzymatic and non-enzymatic antioxidants, further underscoring their critical role in plant development and stress resilience [152,153,154].

The biosynthesis of BRs begins with the precursor campesterol (CR), which undergoes multiple pathways to produce the final active hormone, brassinolide. The BR biosynthetic pathways are categorized into campestanol (CN)-dependent and CN-independent routes. The CN-dependent route is further divided into early and late C-6 oxidation pathways. In the early C-6 oxidation pathway, CN is converted into 6-oxocampestanol (6-oxoCN) and then into castasterone (CS), while in the late C-6 oxidation pathway, CN is transformed into 6-deoxocastasterone (6-deoxoCS) and subsequently into CS. On the other hand, the CN-independent pathway involves the conversion of 22-hydroxycampest-3-one (22-OH-3-one) into intermediates like 6-deoxo-3-dehydroteasterone (6-ddeoxo3DT) and 3-epi-6-deoxocastasterone (3-epi-6-deoxoCT), which then lead to the formation of 6-deoxotyphasterol (6-deoxoTY). The final product across these pathways is BL [155].

The biosynthesis of BRs is tightly regulated by a series of genes that encode enzymes responsible for converting precursors into active BRs that are essential for plant growth and stress responses. Key genes in this pathway include *DWF4*, which encodes a cytochrome P450 monooxygenase involved in the early C-22 hydroxylation step, and *BR6OX1*, which catalyzes the oxidation of 6-deoxocastasterone to castasterone and finally to BL [156]. *CPD* (CONSTITUTIVE PHOTOMORPHOGENESIS AND DWARFISM) and *ROT3* (ROTUNDIFOLIA3) also encode cytochrome P450 enzymes crucial for C-23 and C-3 hydroxylation steps, respectively, in the BR biosynthetic pathways [156]. Additionally, *DET2* (DE-ETIOLATED2) plays a vital role as a steroid 5α-reductase in the conversion of early BR intermediates, while *CYP85A2* catalyzes the final step of converting castasterone to BL [157]. The precise regulation of these genes ensures the appropriate levels of BRs within plants, which is crucial for normal growth, development, and environmental adaptation. Mutations in any of these genes can lead to significant growth abnormalities, such as dwarfism and altered leaf morphology, highlighting their essential role in plant physiology [157,158].

Previous research has demonstrated that ectopic overexpression of TFs like *Atbzr1-1D*, a crucial component of brassinosteroid signaling, can enhance carotenoid accumulation in tomato fruits [159]. Overexpression of DWF4 has been shown to boost vegetative growth in both *A. thaliana* and *B. napus* while influencing plant architecture, seed size, and weight in rice [160,161,162]. Also, the enzyme cytochrome P450 monooxygenase, which DWF4 encodes, catalyzes the C22 α-hydroxylation in the early stages of BR biosynthesis that acts as a rate-limiting factor [163]. BR-deficient or BR-insensitive mutants in *A. thaliana*, pea, and tomato exhibit a range of pleiotropic traits, including dark green leaves, dwarfism, reduced fertility, extended lifespan, and abnormal skotomorphogenesis [164,165]. Although auxin signaling pathways seem unlikely to mediate BR-induced cell elongation in soybean (*Glycine max*) and tomato or BR-inhibited root elongation in *A. thaliana*, studies suggest that IAA levels are reduced in the youngest internodes of BR-insensitive and BR-deficient pea mutants compared to the wild type [166,167]. In rice, BR components like *OsBRI1*, *OsBAK1*, *OsGSK1*, and *OsBZR1* have been identified as orthologs to known *A. thaliana* genes, playing conserved roles in both species. However, rice-specific BR components such as *OsLIC*, *OsDLT*, and *OsTUD1* highlight unique BR functions in rice. The first identified rice BR-insensitive mutant, *d61*, defective in an orthologous gene of *BRI1*, exhibited BR insensitivity, dwarf culms, erect leaves, abnormal skotomorphogenesis, and disorganized microtubule arrangement, underscoring the vital role of BRs in rice development [168,169,170].

Previous studies suggest that BRs enhance drought tolerance in various plants, including *B. napus*, *A. thaliana*, and wheat [171]. Overexpressing the *AtDWARF4* gene in *B. napus* improved drought resistance [172]. Additionally, *A. thaliana* WRKY46, WRKY54, and WRKY70 TFs influence BR-mediated growth and drought response, as the triple mutant (*wrky46 wrky54 wrky70*) shows impaired BR-regulated growth and increased drought tolerance. In tomato, BRs and ABA interact to regulate *RBOH1* expression, NADPH oxidase activity, and apoplastic H_2_O_2_ accumulation, enhancing resistance to oxidative and heat stresses caused by paraquat [173]. Beyond drought tolerance, BRs also contribute to disease resistance, protecting against *Tobacco mosaic virus* (TMV) in tobacco (*Nicotiana benthamiana*) and *Xanthomonas oryzae* and *Magnaporthe grisea* in rice [174,175].

BRs interact with other hormonal pathways and stress-response systems, further amplifying their importance in agriculture. Research on species-specific roles of BR components reveals both conserved and unique functions, emphasizing the role of BR in crop improvement. Advances in genetic engineering, such as the overexpression of DWF4 or Atbzr1-1D, demonstrate the potential of BR to enhance traits like vegetative growth, seed size, and stress resilience, presenting opportunities for improving agricultural productivity and food security.

### 2.7. Jasmonic Acid

Jasmonates are a class of fatty acid derivatives, including critical compounds such as jasmonic acid (JA), methyl jasmonate (MeJA), and jasmonate isoleucine conjugate (JA-Ile) [176]. These molecules, characterized by a core structure of 3-oxo-2-2′-cis-pentenyl-cyclopentane-1-acetic acid, are ubiquitous in higher plants, with exceptionally high concentrations in reproductive tissues and flowers; however, they are present in much lower levels in mature leaves and roots [177]. JAs have evolved into crucial phytohormones in various plant growth and developmental processes. Nevertheless, they were initially recognized as stress-related hormones [177]. Jasmonates modulate numerous physiological activities, including vegetative growth, cell cycle regulation, anthocyanin biosynthesis, and the development of stamens and trichomes [178,179]. They also play vital roles in fruit ripening, stamen growth, root development, senescence, stomatal opening, and the regulation of nutrient uptake, such as that of nitrogen and phosphorus, as well as glucose transport [180,181,182,183,184]. JA is involved in essential processes like regulating metabolites such as phytoalexins and terpenoids [179]. Overall, JAs are integral to regulating plant reproductive growth, nutrient storage, and the movement of assimilates within the plant [185,186].

Jasmonate biosynthesis in plants is a complex process that begins with the release of α-linolenic acid (α-LeA) from chloroplastic galactolipids, initiating a sequential lipid esterification pathway involving chloroplasts and peroxisomes. This pathway is tightly regulated by a series of biosynthetic genes that encode enzymes responsible for converting precursor molecules into active jasmonates, such as JA [187]. The first step in JA biosynthesis is catalyzed by the Lipoxygenase (*LOX*) gene, which encodes an enzyme that oxygenates α-linolenic acid, forming a hydroperoxide derivative. This derivative is then converted into an unstable epoxide by Allene Oxide Synthase (AOS), a crucial enzyme in the pathway. The epoxide is subsequently cyclized by Allene Oxide Cyclase (AOC) to produce 12-oxophytodienoic acid (OPDA), a key intermediate in JA biosynthesis [188]. 12-Oxophytodienoate Reductase 3 (OPR3) further reduces OPDA to form a precursor of jasmonic acid, which undergoes β-oxidation catalyzed by Acyl-CoA Oxidase (ACX) to produce JA [189]. Finally, Jasmonate Resistant 1 (*JAR1*) encodes an enzyme that conjugates JA with isoleucine, forming the bioactive jasmonoyl–isoleucine (JA-Ile), which is essential for JA signaling [190,191]. The JA biosynthesis process also involves spontaneous hydrolysis, which can lead to the formation of α- and ÿ-ketols and non-enzymatic cyclization to racemic OPDA. These non-enzymatic reactions must be considered when assaying enzyme activity and quantifying JA and OPDA levels [192]. The overall biosynthesis and metabolism of JA are represented schematically by highlighting the intricate coordination between enzymatic and spontaneous reactions in this essential plant signaling pathway [192].

Sweet wormwood (*Artemisia annua*) accumulates higher JA levels under cold stress, with JA biosynthetic genes being activated in response, suggesting that JA plays a role in cold stress tolerance, as exogenous JA treatment further enhanced tolerance [193]. Similarly, in sweet orange (*Citrus sinensis*), JA application increased antioxidant gene expression and reduced H_2_O_2_ levels, improving cold stress resistance [194]. JA accumulation also occurs under drought stress, as observed in wheat, where JA levels increased fivefold after 24 h of drought. Additionally, drought stress upregulated JA biosynthesis genes like *LOX1*, *AOS1*, *AOC1*, and *OPR3*, indicating the involvement of JA in drought response [195,196]. In *A. thaliana*, *ABCG6* and *ABCG20* (also known as JAT3 and JAT4, respectively) play crucial roles in regulating the translocation of JA between leaves, particularly in response to wounding. These phloem-expressed, plasma membrane-localized transporters work together, with high affinity for JA and low affinity for JA-Ile, to facilitate long-distance JA movement, ensuring coordinated defense responses throughout the plant [197,198].

The multifunctional roles of JAs in plant growth, development, and stress responses highlight their potential for agricultural applications. By modulating JA biosynthesis or transport pathways, we can enhance crop resilience to environmental stresses, improve nutrient uptake efficiency, and optimize fruit ripening and reproductive growth processes.

### 2.8. Salicylic Acid

Salicylic acid (SA) is a crucial phenolic compound known as 2-hydroxy benzoic acid and is vital in regulating various biochemical and physiological processes in plants [199,200,201]. The basal levels of SA can vary significantly, with up to a 100-fold difference among plants of the same family and notable variation between species [202,203]. SA levels are mainly known to increase in response to infections by viruses, fungi, insects, and bacteria, triggering a strong defense response in the host [204,205,206].

One of the key defense mechanisms involving SA is systemic acquired resistance (SAR), which is associated with activating pathogenesis-related (*PR*) genes. During pathogen attacks or exposure to abiotic stressors, such as salt, drought, extreme temperatures, or high light intensity, SA accumulates in both infected and systemic tissues [207]. This accumulation leads to upregulating numerous genes, including those involved in oxidative stress protection and the production of PR proteins [208]. In addition to its role in defense signaling, SA is closely linked to various aspects of plant growth and development. It influences photosynthesis, transpiration, ion uptake, thermogenesis, and senescence while interacting with other hormones, showcasing its multifaceted role in plant biology [209].

Two key pathways have been identified as the primary regulators of SA biosynthesis in plants: the isochorismate (ICS) pathway and the phenylalanine ammonia-lyase (PAL) pathway [210,211]. While both pathways operate simultaneously in plants, the ICS pathway is the predominant route, responsible for more than 90% of SA production [211,212]. In the ICS pathway, the first step involves the conversion of chorismate to isochorismate by the isochorismate synthase enzyme [213]. Isochorismate is synthesized in plastids and transported to the cytosol via the *EDS5* (Enhanced Disease Susceptibility 5) gene, which encodes a plastidal MATE transporter enzyme [210,211]. Once in the cytosol, isochorismate is conjugated with L-glutamate by the AvrPphB Susceptible 3 (PBS3) enzyme, forming isochorismate-9-glutamate (ICS-Glu). This intermediate spontaneously decomposes into SA and 2-hydroxy-acryloyl-N-glutamate, with occasional involvement from an acyltransferase encoded by the *EPS1* (Enhanced Pseudomonas Susceptibility 1) gene. Notably, the activity of the PBS3 enzyme is subject to negative feedback regulation by SA itself [211,214]. The PAL pathway is secondary, accounting for about 10% of SA synthesis. In this pathway, the PAL enzyme converts phenylalanine to trans-cinnamic acid (t-CA), which is subsequently converted to SA via intermediates such as ortho-coumaric acid and benzaldehyde. The gene encodes the PAL enzyme in multiple copies within plants, ensuring the pathway contributes to overall SA levels. Together, these pathways and their associated genes ensure the effective biosynthesis of SA, which is crucial for plant defense mechanisms, growth, and development [215].

SA is also recognized as an internal signaling molecule that interacts with ROS in specific signal transduction pathways. For example, exogenous SA treatment has been shown to induce the generation of H_2_O_2_ in *A. thaliana*, tobacco, and mustard (*Brassica nigra*). Both SA and H_2_O_2_ can enhance the tolerance of potato (*Solanum tuberosum*) microplants to high temperatures, suggesting that SA and H_2_O_2_ share common signaling pathways [216,217]. Several *A. thaliana* mutants that over accumulate SA, such as *acd6*, *cpr5*, and *siz1*, exhibit a dwarf phenotype characterized by shorter stems, smaller leaves, and reduced floral organs compared to the wild type [218,219]. Evidence suggests that SA negatively affects certain cellular processes in *A. thaliana* leaves. For example, SA-deficient *NahG* transgenic plants showed a higher growth rate than wild-type plants, with a 1.7-fold increase in leaf rosette biomass during the early reproductive stage [220]. Transcriptome analysis in plants has identified the role of SA signaling in regulating heat stress-responsive genes during thermotolerance. These include *NPR1* (nonexpresser of pathogenesis-related genes), *HSP*s, *MBF1c* (multiprotein bridging factor 1c), TGA, and *PR-1* [221,222]. The external application of SA has been found to promote the production of HSPs in species such as *A. thaliana*, tomato, and rice [223,224,225,226].

During drought stress, SA levels rise in various plant species, including barley (*Hordeum vulgare*), green olive (*Olea europaea*), and sage (*Salvia officinalis*) [227,228,229]. SA contributes to drought resistance by strengthening the antioxidant system, improving water retention, increasing proline content, and interacting with other phytohormones [230]. Pre-treatment with SA has been shown to reduce drought-induced oxidative stress by enhancing the expression of redox-regulating genes and stimulating proline synthesis-related pathways [231]. Studies indicate that SA exposure can mitigate chilling injuries in the seed germination of mountain rye (*Secale strictum*), muskmelon (*Cucumis melo*), and bean plants [232,233,234]. This protective mechanism may be due to the ability of SA to stimulate protein synthesis, such as via the 20S proteasome, and enhance enzyme activities involved in metabolic pathways like the pentose phosphate pathway, gluconeogenesis, and glycolysis, which help plants transition from a dormant state to active growth [235].

SA is an essential phytohormone that plays a key role in plant immune responses against pathogens. However, many clubroot pathogens specifically target SA by interfering with its production, accumulation, or downstream signaling pathways [236]. In apple, SA notably enhances the expression of *NPR1* and *PR1* while also increasing internal SA levels [237]. Previous research has highlighted that the SA signaling pathway is responsible for producing antimicrobial PR proteins, which are crucial for defending plants against bacterial pathogens [238]. Similarly, another study indicated that bacterial infections in plants trigger the release of SA-dependent PR proteins, aiding in establishing SAR through SA signaling [239]. Adverse conditions like water scarcity and nutrient deficiency can cause plant damage similar to that inflicted by pathogens. For instance, in house leek (*Sempervivum tectorum* L.), the absence of visible pathogens is noted, and SA levels are strongly linked to leaf hydration and relative water content [240]. Moreover, in table grapes (*Vitis vinifera* L.), the interaction between phytohormones such as ABA, IAA, SA, and JA plays a key role in fruit ripening and defense against necrotrophic fungal pathogens [241].

Overall, SA serves as a pivotal molecule in enhancing plant defense and resilience, making it a valuable tool for sustainable agricultural practices. By leveraging its role in SAR and its interaction with ROS, we can improve crop tolerance to biotic and abiotic stresses. Furthermore, understanding the complexities of SA biosynthesis pathways provides opportunities for targeted interventions to support plant growth and stress adaptation.

### 2.9. Strigolactone

Strigolactones (SLs) are a class of carotenoid-derived terpenoid lactones, primarily isolated from plant root exudates, that have emerged as a newly defined group of phytohormones [242,243,244,245]. These compounds play crucial roles in regulating plant architecture and enhancing resilience against adverse environmental conditions such as drought, salt stress, and fungal infections [246,247]. In addition to their protective functions, SLs influence various aspects of plant development, including regulating natural branching patterns, stimulating seed germination, and shaping both above-ground structures like leaves and below-ground structures like roots. SLs also enhance photosynthetic efficiency and delay leaf senescence by showing multifaceted roles in plant growth and physiology [248,249].

Strigolactone biosynthesis begins in the chloroplasts. During its biosynthesis, the DWARF27 (D27/β-carotene isomerase) enzyme requires iron as a cofactor, which catalyzes the isomerization of all-trans-β-carotene into 9-cis-β-carotene [250,251]. This initial step is followed by successive catalytic processes mediated by carotenoid cleavage dioxygenases 7 and 8 (MAX3 and MAX4), producing carlactone (CL) [252]. Carlactone is then transported to the cytoplasm, where it undergoes further oxidation by the cytochrome P450 711A (CYP711A) family, resulting in carlactonoic acid (CLA) [250,253]. CLA can subsequently be converted into two types of SLs: canonical SLs, which feature a tricyclic lactone structure composed of three rings (ABC rings), and noncanonical SLs, which lack these typical ABC rings [254,255,256].

As signaling molecules in the rhizosphere, SLs exhibit diverse biological activities of significant agricultural interest, mainly due to their regulatory roles under biotic and abiotic stress conditions [257]. Despite extensive research efforts, only a limited number of SL-responsive genes have been identified, including BRANCHED 1 (*BRC1*) in *A. thaliana* and pea, *MAX3*, *MAX4*, *SMXL2, 6, 7,* and *8*, *STH7/BZS1, DLK2, KUF1,* and several auxin-responsive genes in *A. thaliana* [258,259]. In rice, notable SL-responsive genes include *D53* and *OsCKX9*, which are involved in cytokinin metabolism [260,261]. The regulation of salt stress tolerance by SLs in *Sesbania cannabina* seedlings is mediated by H_2_O_2_ produced through NADPH oxidase activity [262]. GR24, a synthetic SL analog, is a positive stress response regulator. Exogenous application of GR24 has been shown to improve drought and salt tolerance in *A. thaliana*, promote growth and photosynthesis, alleviate oxidative stress, and enhance salt stress tolerance in crops like rapeseed (*Brassica napus*) [263]. GR24 increases photosynthesis, antioxidant enzyme activity, and gene expression in tomato seedlings, thereby improving tolerance to low light conditions [264].

Studies on tomato plants with SL biosynthetic mutants have shown that SLs play a beneficial role in plant defense against fungal pathogens like *Botrytis cinerea* and *Alternaria alternata*, as well as root-knot nematodes such as *Meloidogyne incognita*. Interactions between SLs and other plant hormones likely influence this protective effect [265,266,267].

Using synthetic SL analogs such as GR24 offers a practical approach to improving stress tolerance, boosting photosynthetic efficiency, and regulating plant architecture. Targeted manipulation of SL biosynthesis or signaling pathways could lead to innovative strategies for sustainable crop improvement and stress management.

## 3. Transcription Factors

Transcription factors are a diverse group of regulatory proteins that play a crucial role in the transcription regulation of target genes. The DNA-binding domains of TFs bind to specific DNA regulatory sequences, initiating or repressing the transcription of their target genes. When a TF binds to a motif within a promoter sequence, it can either promote or inhibit transcription initiation, depending on its functional domains and interacting partners [268]. The plant world consists of a vast array of TFs. *A. thaliana* has 2300 TFs, corresponding to nearly 8.3% of the total genes. Likewise, major crop species show similar percentages, such as 6.5% in rice, 5.7% in wheat, and 6.1% in canola (*B. napus*) [269,270,271,272].

TFs are crucial in mediating plant signaling pathways and determining how plants react to abiotic and biotic triggers. They also participate in the reaction to internal cues of plants, coordinating the collaboration between various components during developmental stages [273]. Upon perceiving various environmental cues, these TFs undergo transcriptional- and translational-level alterations and ultimately prepare the plant for the stresses by modulating target genes [274,275,276]. This review focuses on common TFs such as NAC, HSF, WRKY, AP2/ERF, bZIP, MADS-box, and the MYB family, which make a massive contribution to withstanding abiotic and biotic stresses as well as the growth and development regulation of plants.

### 3.1. NAC

The NAC (NAM—no apical meristem, ATAF—*A. thaliana* transcription activation factor, and CUC—cup-shaped cotyledon) transcription factor was first reported in 1997, and its significance lies in its active involvement in regulating plant growth developmental processes and orchestrating the reactions of plants when faced with environmental challenges [277,278]. Being a sophisticated family exclusive to plants and ranking as the fourth largest group of TFs in the plant family, the NAC TFs exhibit their presence across a diverse array of plant species. While the quantity of NAC TF members varies throughout numerous species, their essential structural characteristics tend to share commonalities [279].

A typical NAC TF comprises a relatively unchanging NAC domain and a more diverse transcription regulatory region (TRR) [280]. This NAC domain is positioned at the end of the N-terminus of the protein and typically encompasses around 150 amino acid residues [280]. NAC TFs frequently exert their functions as dimers, and the location for dimerization is also situated within the NAC domain [281]. The NAC domain can be divided into five distinct subdomains, designated as A through E. Among these, subdomain A contributes to creating an effective dimeric structure. Subdomains C and D, which are positively charged and have vital conservation, tend to serve as binding sites for DNA. As for subdomains B and E, their roles might involve linking with the varied functions exhibited by NAC TFs [281]. Within the NAC domain, single or dual-core nuclear localization signals exist, with crucial involvement of lysine residues found in subdomain D for moving into the nucleus [282]. Additionally, the NAC domain exerts control over protein binding, which is a factor that can determine the function and destiny of the NAC proteins. This control is particularly pivotal in interactions between plant–pathogen interactions or in conferring resistance to stresses [277].

These NAC proteins are functional factors across virtually all plant growth and development phases, as well as biotic/abiotic responses. As in the development of the plant, the roles of NAC TF encompass diverse processes, including cell division, the creation of secondary cell walls, the establishment of shoot apical meristems, the development of floral organs, fruit maturation, and the aging process of leaves [283,284,285].

NAC extends its function in abiotic/biotic stresses into the plant’s innate immune system, basal defense, and systemic acquired resistance [286]. Many NAC genes play crucial roles in plant responses to abiotic stresses like drought and salinity (Table 1). In transgenic rice, *Os01g66120/OsNAC2/6* and *Os11g03300/OsNAC10* have been shown to improve drought and salt tolerance. Similarly, *Os03g60080/SNAC1* was found to increase grain yield by 21–34% under drought stress [287]. The *A. thaliana ANAC092* gene mediates complex regulatory networks involved in salt-induced senescence and seed maturation [288]. Promoter–GUS fusion studies in rice and *A. thaliana* have revealed that genes such as *Os01g66120/SNAC2/6*, *Os11g03300/OsNAC10*, *RD29A*, *COR15A*, *KIN1*, and *COR6.6* are regulated during plant development in roots, leaves, and pollen under both stress (drought, cold) and normal conditions [289].

Overexpression studies further highlight that the plants overexpressing *GmNAC085* exhibit enhanced drought resistance, while the overexpression of *GmNAC11* increases sensitivity to salt and mannitol stresses [290,291]. Moreover, microarray analysis of drought-treated rice revealed that 17 NAC genes are induced under both severe and mild drought conditions [292]. NAC proteins also respond to extreme temperatures. In rice, the NAC transcription factor *ONAC063* is activated by high-temperature stress [293]. Similarly, transgenic *A. thaliana* plants overexpressing *ANAC042* show improved heat stress tolerance compared to wild-type plants [294]. The overexpression of *ZmSNAC1* enhances tolerance to both drought and low temperatures [295]. Additionally, several NAC genes, including *OsNAC10*, *TaNAC4*, *NTL6*, and others, are upregulated in response to low temperatures [296,297,298]. Conversely, heat stress specifically induces a CsNAM-like protein gene in tea (*Camellia sinensis*) plants [299].

Among biotic stresses, NAC TFs have been linked to defense mechanisms against powdery mildew and stripe rust in wheat. Previous studies have identified *TaNAC6* as a key factor in enhancing resistance to *Blumeria graminis* f. sp. *tritici* (Bgt), with its overexpression in transgenic plants significantly improving defense [300]. Similarly, *TaNAC8* plays a positive role in protecting wheat from stripe rust caused by *Puccinia striiformis* f. sp. *tritici* (Pst), with its expression detected 24 h post-inoculation during an incompatible interaction [301]. In contrast, *TaNAC30*, *TaNAC21*, and *TaNAC22* negatively regulate wheat resistance to *Pst* [302,303].

In *A. thaliana*, overexpression of *ANAC019* or *ANAC055* reduces resistance to *Botrytis cinerea* [304]. However, the NAC TF *ATAF1* positively regulates resistance to the biotrophic fungus *Blumeria graminis* f. sp. *graminis* (Bgh) while weakening resistance to other pathogens, such as *Pseudomonas syringae*, *Botrytis cinerea*, and *Alternaria brassicicola* [305]. Additionally, *NAC4* is negatively regulated by *microRNA164* in *A. thaliana*, which enhances hypersensitive response (HR)-mediated cell death against avirulent bacterial pathogens like *Pseudomonas syringae* pv. *tomato* DC3000 [306].

In rice, *OsNAC066* positively regulates resistance to *Magnaporthe grisea* by modulating the ABA signaling pathway and influencing amino acid and sugar accumulation [307]. Likewise, *OsNAC111*, *ONAC122*, and *ONAC131* contribute to defense against *M. grisea* [308,309]. Overexpression of *OsNAC58* further enhances resistance to both *Magnaporthe oryzae* and bacterial blight caused by *Xanthomonas oryzae* pv. *oryzae* [309].

NAC TFs are also involved in phytohormone-mediated defense signaling. In tomato, *SlNAP1* enhances pathogen resistance by activating genes involved in GA inactivation and the biosynthesis of SA and ABA [310]. Similarly, in grapes, *VvNAC72* strengthens resistance to downy mildew by inhibiting *VvGLYI-4* expression, leading to methylglyoxal accumulation and increased ROS levels, which ultimately activate the SA-mediated defense pathway [311]. In rice, *OsNAC19* functions in JA signaling, while *ONAC122* and *ONAC131* regulate defense responses by modulating genes such as *OsLOX*, *OsPR1a*, *OsWRKY45*, and *OsNH1*. In *A. thaliana*, *ANAC019* and *ANAC055* contribute to JA-mediated defense by activating JA-responsive genes and cooperating with *AtMYC2*. Similarly, *SlNAP1* in tomato enhances stress resistance by promoting GA inactivation while stimulating SA and ABA biosynthesis [310]. In banana (*Musa acuminata*), *MaNAC5,* and in *A. thaliana*, *ANAC019* and *ANAC055,* function within the JA signaling pathway [312,313].

Additionally, *OsNAC066* in rice boosts resistance to rice blast and bacterial blight by suppressing the ABA pathway, leading to increased soluble sugar and amino acid accumulation [306]. In cotton (*Gossypium hirsutum* L.), *GhORE1* influences pathogen resistance by inhibiting ethylene synthesis [314].

While significant progress has been made in understanding NAC TF functions, further research is needed to unravel their intricate regulatory networks, identify novel members with potential agronomic applications, and harness their capabilities for crop improvement. Given their broad regulatory scope, NAC TFs remain a promising target for genetic engineering and breeding strategies aimed at enhancing plant tolerance to environmental stresses and improving agricultural productivity.

### 3.2. HSF

Heat shock factor (HSF) was initially identified in yeast as a transcription factor that specifically binds to the promoters of heat shock protein (HSP) genes [315]. During summer, terrestrial systems often experience mild to severe heat stress from midday until late afternoon [316]. To ensure the survival of the plants, a slight temperature rise early in the morning must be detected to initiate an appropriate genetic response. This response involves upregulating specific genes and synthesizing HSPs to confer acquired thermotolerance [317]. Although HSPs were initially identified for their role in thermotolerance, they were later found to be involved in various abiotic stress responses and normal plant developmental processes [318]. In response to stresses, the transcriptional regulation of HSPs, known as heat shock response (HSR), is activated [319]. The HSR is controlled by heat shock factors (HSF), which bind to specific *cis*-acting elements in the promoter region, known as heat shock elements (HSEs) [320,321].

Under heat stress, transcriptome and proteome analyses have revealed regulatory responses involving HSPs [322,323,324]. All eukaryotic organisms synthesize HSPs, many belonging to the conserved chaperone families. A conserved subfamily of these proteins, termed “heat-induced molecular chaperones”, includes HSP100s, HSP90s, HSP70s, HSP60s, HSP40s, and HSP20s [325,326]. These chaperones are 20 times more likely to be induced by heat compared to non-chaperone proteins [327,328]. Among them, HSP20s are the most heat-responsive due to their significant induction in plants [323].

The accumulation of HSPs in land plants is triggered by a signal originating from the plasma membrane that ultimately leads to the activation of HSF families [328,329]. HSFs are classified into three families, HSFA, HSFB, and HSFC, which have different functions from each other. Under regular conditions, the activity of HSFA is negatively regulated by HSP90 and maintained in the form of phosphoproteins [330]. During the initiation of stress, this regulation is reversed, and HSP90 starts to dissociate and changes into a functional trimer, which then binds to the HSE in the promoter region, after which transcription takes place and HSPs are synthesized [331,332].

In *A. thaliana*, overexpression of HSFA1, HSFA2, HSFA3, and HSFA4A improves tolerance to heat and other stresses, including drought, salinity, submergence, anoxia, Cd toxicity, high light, and oxidative stress [333,334,335,336,337]. However, a previous study has shown that tomato HSFA1a is a master regulator for acquired thermotolerance, with a unique, non-redundant role [338]. Similarly, overexpression of soybean *GmHSFA1* enhances thermotolerance in transgenic soybeans by activating downstream HSP genes, such as *GmHsp70* and *GmHsp22* [339].

Overexpression of *VpHSF1* from Chinese grape (*Vitis pseudoreticulata*) in tobacco reveals a dual role, acting as a negative regulator of basal thermotolerance while positively influencing acquired thermotolerance [340]. Additionally, *A. thaliana HSFA1b* enhances thermotolerance by upregulating key heat-responsive genes like *HSP70* and *HSP90* and regulating *OPR3*, which is a gene upstream in the jasmonic acid pathway. JA-mediated thermotolerance is linked to increased expression of *DREB2A*, which is reduced in *opr3* mutants [341]. Moreover, *A. thaliana* HSFA1b enhances thermotolerance in potato (*Solanum tuberosum* L.) by upregulating the expression of heat shock protein genes HSP70 and HSP90 [342].

Beyond heat stress, HSFs are involved in responses to other abiotic stresses. In *A. thaliana*, HSFA1s contribute to seedling tolerance to salt, osmotic, and oxidative stresses [343]. Overexpression of chickpea (*Cicer arietinum*) *CarHSFB2* in *A. thaliana* enhances drought tolerance by increasing the transcript levels of stress-responsive genes such as *RD22*, *RD26*, and *RD29A* during the seedling stage under drought conditions [344].

These findings underscore the critical regulatory roles of HSFs in improving plant adaptability to a wide range of environmental challenges through their involvement in stress-responsive pathways and gene activation.

### 3.3. WRKY

Among the most prominent families of transcriptional regulators in plants [345], the WRKY TF family was first characterized when the WRKY gene SPF1 was cloned from sweet potato (*Ipomoea batatas*) [346], followed by identifying numerous other WRKY members [347,348,349]. The exposure of plants to various stresses, including oxidative stresses, extreme temperatures, drought, and salinity, poses a global threat to key crops, significantly affecting plant growth and productivity [350,351]. The WRKY family constitutes a significant group of TFs widely distributed in plants, playing vital roles in various developmental and physiological processes and stress responses [352].

WRKY TFs contain a WRKY protein domain, which has a 60-amino-acid-long DNA binding domain (DBD), characterized by an invariant heptad WRKYGQK (hence called WRKY) amino acid motif at the N-terminus and a zinc-binding motif at the C-terminus [353]. The WRKY TF family is classified into three major groups based on the number of WRKY domains and the type of zinc finger motifs: I, II, and III [354]. Several WRKY proteins are placed in group I, which has two WRKY domains and a C2H2-type zinc finger motif at either the C-terminus (CT) or the N-terminus (NT). Groups II and III members possess only one WRKY domain at the CT. Group II is further divided into five subgroups (IIa, IIb, IIc, IId, and IIe). Group III members uniquely contain the C2HC-type zinc finger. These domains and motifs are crucial for WRKY proteins to bind to the W-box (TTGACT/C) in the promoter of downstream target genes [355], inducing gene expression to maintain cellular homeostasis [345,356]. WRKY proteins regulate gene expression by specifically binding to cis-regulatory elements in the 5′ upstream region of the gene [357]. Multiple W-boxes are found in the promoter regions of structural genes and WRKY genes themselves [358].

Under stress conditions, plants trigger molecular, cellular, and physiological changes, including stomatal closure, reduced photosynthesis, increased osmolality, and the activation of numerous stress response genes [359,360,361]. Across the various plant species, the expression of WRKY genes plays a pivotal role in response to these conditions, helping plants to adapt [362,363]. Genomic and transcriptomic studies have helped to understand the whole genomics version of WRKY genes in different plant species and to reveal the mode of action of WRKY TFs in plant stress responses [353].

In Chinese white pear (*Pyrus communis*), *PbrWRKY53* enhances drought tolerance by binding to and activating the expression of *PbrNCED1*, which stimulates vitamin C biosynthesis [364]. Similarly, in iris (*Iris germanica*), *IgWRKY32* and *IgWRKY50* work together to boost ABA signaling, thereby improving the resistance to drought stress [365]. However, in cotton, *GhWRKY21* and *GhWRKY33*, as well as *OsWRKY5* in rice, suppress ABA signaling, leading to diminished drought tolerance [366,367,368]. Additionally, *OsWRKY55* in rice negatively affects drought resistance by activating OsAP2-39, which reduces ethylene synthesis [369].

In other plants, WRKY factors play roles in salt and cold stress responses. Overexpression of *GarWRKY17* and *GarWRKY104* enhances salt tolerance in *A. thaliana* across different developmental stages [370]. Similarly, *PeWRKY83* improves salt tolerance in *A. thaliana* by increasing proline accumulation, germination rates, and membrane stability under salt stress [371]. However, some WRKYs act as negative regulators, too. For instance, *PalWRKY77* in poplar (*Populus alba* L.) compromises salt resistance by suppressing ABA-responsive genes [372], and the maize (*Zea mays*) *ZmWRKY20-ZmWRKY115* complex inhibits the expression of *ZmbZIP111*, heightening salt sensitivity in seedlings [373]. For cold tolerance, *CdWRKY2* in bermudagrass (*Cynodon dactylon*) enhances responses by activating *CdSPS1* and *CdCBF1*, promoting sucrose biosynthesis and the CBF pathway [374]. In contrast, rice *OsWRKY63* negatively regulates cold tolerance by downregulating genes involved in ROS scavenging and chilling responses [375].

WRKY TFs also influence nutrient management. Suppressing *OsWRKY28* in rice reduces phosphate (Pi) accumulation, while *OsWRKY21* and *OsWRKY108* positively regulate *OsPHT1;1*, facilitating Pi uptake [376,377]. In apple (*Malus domestica*), overexpression of *MdWRKY39* increases phosphorus deficiency by upregulating *MdPHT1;7* [378]. Meanwhile, in poplar, a phosphate starvation response protein (PHR1) interacts with *PtoWRKY40* to inhibit *PtoPHT1*, thereby increasing the Pi content [379].

In barley (*Hordeum vulgare*), *HvWRKY1* and *HvWRKY2* are activated by the MAMP flg22 and act as repressors of PTI against the powdery mildew fungus *Blumeria graminis* f. sp. *hordei*. However, the fungal effector AVRA10 triggers a specific interaction between the resistance protein MLA10 and *HvWRKY1/2*, leading to the inactivation of their repressor function [380]. In *A. thaliana*, *AtWRKY18*, *AtWRKY40*, and *AtWRKY60*, homologs of *HvWRKY1* and *HvWRKY2*, also negatively regulate PTI against *Pseudomonas syringae* and the powdery mildew fungus *Golovinomyces orontii* [381,382]. Similarly, in rice, the *OsWRKY62* gene acts as a negative regulator of both PTI and ETI mediated by the *Xa21* gene to *Xanthomonas oryzae* [382].

In *A. thaliana*, *AtWRKY3* enhances resistance to the necrotrophic fungus *Botrytis cinerea*, while *AtWRKY4* protects against both necrotrophic and biotrophic pathogens. However, overexpression of *AtWRKY3* and *AtWRKY4* suppresses pathogen-induced *PR1* expression [383]. Similarly, *AtWRKY33* acts as a positive regulator against *Alternaria brassicicola* and *B. cinerea* by activating defense-related genes, including those involved in JA signaling [384].

WRKY TFs also influence hormonal signaling pathways. For instance, overexpression of *AtWRKY18* and *AtWRKY70* in *A. thaliana* enhances the expression of defense-related genes, including SA-induced *PR1* [385]. In contrast, the increased susceptibility of the *atwrky33* mutant to *B. cinerea* is associated with SA-mediated repression of the JA pathway [386]. *AtWRKY28* and *AtWRKY75* contribute to SA- and JA/ET-dependent defenses, improving resistance to oxalic acid and fungal infections [387]. In contrast, *AtWRKY57* suppresses immunity against *B. cinerea* by competing with *AtWRKY33* for binding to SIB1, SIB2, JAZ1, and JAZ5, thereby altering the JA-mediated defense signaling pathway [388].

In paprika (*Capsicum annuum*), *CaWRKY27* positively regulates resistance to *Ralstonia solanacearum* by modulating SA-, JA-, and ET-mediated signaling pathways in *Nicotiana tabacum* [389]. Likewise, *WRKY8* in *A. thaliana* influences susceptibility to *Pseudomonas syringae* and *B. cinerea*, while also mediating ABA-ET crosstalk to enhance resistance against *TMV-cg* [390,391].

Some WRKY TFs act as negative regulators of plant defense. *AtWRKY18* and *AtWRKY40* suppress resistance against the biotrophic pathogen *Golovinomyces orontii*, which causes powdery mildew [392]. In *A. thaliana*, *AtWRKY38* and *AtWRKY62*, belonging to group III WRKY TFs, negatively regulate defense against *P. syringae*, with disease resistance significantly enhanced in their single and double mutants [393].

In wheat, *TaWRKY70* plays a positive role in high-temperature seedling plant (HTSP) resistance to *Puccinia striiformis* f. sp. tritici (Pst), likely through SA and ET signaling activation [394]. Additionally, WRKY TFs are involved in mitogen-activated protein kinase (MAPK) signaling, which regulates stress-induced defenses. *AtWRKY22* and *AtWRKY29* are key components of MAPK-mediated defense responses, with transient expression of *AtWRKY29* enhancing resistance to *P. syringae* [395]. Beyond hormonal pathways, WRKYs contribute to plant immunity through mechanisms such as small RNA modulation, epigenetic regulation via histone methylation, proteasome-mediated protein degradation, and inter-organelle retrograde signaling [396,397].

These findings illustrate the complex roles of WRKY TFs in balancing immunity and hormonal signaling to mediate plant responses to pathogens and the multifaceted roles of WRKY TFs in managing plant stress responses and nutrient homeostasis.

### 3.4. AP2/ERF

APETALA2/ethylene-responsive element (AP2/ERF) binding factors are critical for regulating plant responses to abiotic stress and improving crop yields, and they make up one of the most evolutionarily ancient and expansive groups of TFs in plants. They are vital in controlling various developmental processes and are central to hormonal regulation and stress responses from a signaling perspective [398,399,400,401,402].

The AP2/ERF superfamily is characterized by the presence of an AP2 DNA-binding domain, approximately 60 amino acids in length, which directly interacts with cis-acting elements such as dehydration-responsive elements (DRE)/C-repeat elements (CRT) and/or the GCC-box at the promoters of target genes [400,401,403]. This superfamily is further classified into four significant subfamilies: DREB (Dehydration-Responsive Element-Binding), ERF (Ethylene-Responsive Element-Binding protein), AP2 (APETALA2), and RAV (Related to ABI3/VP), along with a few unclassified factors known as Soloists [401,402,404].

AP2/ERF was first identified in *A. thaliana* by Jofuku in 1994 and has been extensively researched in various plant species [405]. So far, hundreds of AP2/ERF members have been identified in higher plants. For instance, 147, 163, and 131 AP2/ERF TFs have been reported in *A. thaliana*, rice, and cucumber (*Cucumis sativus*) [406,407], respectively. In *Brassica rapa* ssp. and *Pekinensis*, 65 and 291 members were identified based on EST and genome sequences, respectively [408,409]. Additionally, 148, 167, 53, and 117 AP2/ERF TFs have been reported in soybean, maize, barley, and common wheat [410,411,412]. In *Brassica juncea*, multiple copies of the AP2 gene were identified, indicating genetic diversity in this tetraploid species [413].

AP2/ERF TFs are involved in plant growth and development, including root initiation and development, leaf size, floral patterning, floral meristem establishment, flower senescence, seed development, grain development, fruit development and ripening, and spikelet meristem fate [414,415,416,417,418,419,420,421]. They are also vital in plant hormone signal transduction pathways, which aid in modulating processes like plant height, root and flower development, and seed germination [399,400]. Moreover, these factors have shown their function in regulating responses to abiotic stresses such as heat, cold, salinity, drought, and osmotic stress, as well as in biotic stresses such as microbial and herbivore insect attacks [422,423]. Furthermore, the genetically modified plants that enhanced AP2/ERF expression have shown elevated resistance to biotic and abiotic stresses [424,425,426].

In rice, several AP2/ERF TFs act as positive regulators of stress tolerance. *OsDREB1A* promotes cold tolerance by activating the ion channel gene *OsCNGC9*, which enhances extracellular calcium influx and cold stress-related gene expression [427]. Similarly, *OsDREB1BI* responds to both high and low temperatures, conferring cold and heat tolerance when overexpressed in *A. thaliana* and tobacco [428]. Overexpression of *OsDREB1D* and *OsDREB1E* improves cold and salt tolerance in transgenic *A. thaliana*, while transgenic rice and *A. thaliana* carrying the *OsDREB1F* gene exhibit enhanced resistance to high salinity, drought, and low temperatures [429].

AP2/ERF TFs play a critical role in modulating plant responses to various hormones, including ethylene, ABA, JA, and redox signaling [430]. For instance, *AtERF11* in *A. thaliana* regulates gibberellin biosynthesis by upregulating the expression of *GA3ox1* and *GA20ox* genes, influencing GA signaling pathways [431]. *OsDREB2B* is another AP2/ERF TF that significantly improves drought tolerance in rice [432]. However, its overexpression results in reduced plant height, a phenotype that can be mitigated by exogenous GA3 application [433]. Additionally, overexpression of *ZmEREB20* in *A. thaliana* increases ABA sensitivity, delays seed germination under salt stress, and regulates genes associated with ABA and GA signaling [434]. Moreover, the drought-responsive ERF gene *OsDERF1* negatively regulates ethylene synthesis by activating the transcription of *OsERF3* and *OsAP2-39*. This regulation contributes to a negative role in drought stress response [433].

AP2/ERF TFs play essential roles in plant defense by regulating gene expression in response to pathogens. Their functions vary from positive regulation of immunity to acting as transcriptional repressors, influencing SA-, JA-, and ET-mediated defense pathways.

In rice, *OsERF922* negatively regulates resistance to *Magnaporthe oryzae* by suppressing defense-related genes [435]. In contrast, overexpressing *OsERF83* significantly reduces lesion formation caused by *M. oryzae*, indicating its positive role in rice blast resistance [436]. Similarly, in soybean, *GmERF113* enhances resistance to *Phytophthora sojae* by activating *GmPR1* and *GmPR10-1* expression [437]. In tomato, silencing *StERF3* upregulates *PR1*, *NPR1*, and *WRKY1*, leading to increased immunity against *Phytophthora infestans* [438].

Some AP2/ERF TFs function as transcriptional repressors. For instance, *AtERF9* binds to the GCC-box of the *PATHOGEN-INDUCIBLE PLANT DEFENSIN (PDF1.2)* gene in *A. thaliana*, repressing its expression. Knocking out *AtERF9* significantly enhances *PDF1.2* levels, improving resistance to *Botrytis cinerea* [439].

AP2/ERFs regulate plant immunity by modulating SA biosynthesis and signaling. In apple, *MdERF11* enhances resistance against *Botryosphaeria dothidea* by promoting SA production [440]. Similarly, *ERF11* is induced upon SA treatment and *Pseudomonas syringae* (Pst) DC3000 infection, and its disruption suppresses SA-mediated defenses, decreasing resistance to *Pst DC3000* [441].

AP2/ERFs also regulate JA/ET-dependent immunity. Overexpression of *ERF5* or *ERF6* in *A. thaliana* enhances resistance to *B. cinerea* by upregulating JA/ET-responsive genes [442]. ORA59 further promotes JA/ET signaling by directly binding to the GCC-boxes of *PDF1.2*, boosting immunity against *B. cinerea* [443,444].

Some AP2/ERF TFs regulate multiple hormone pathways simultaneously. In rice, OsERF3 enhances resistance to herbivores by increasing the accumulation of SA, JA, and ET [445]. Likewise, in tomato, SlERF2 boosts SA and JA levels, enhancing immunity against *Stemphylium lycopersici* [446].

Harnessing the potential of the AP2/ERF transcription factor family offers significant opportunities for future agricultural advancements. Researchers can develop stress-tolerant, high-yielding plant varieties tailored for challenging environments by leveraging their role in stress regulation and crop development. Continued exploration of their genetic diversity and specific functions across plant species will pave the way for innovative solutions in sustainable crop improvement.

### 3.5. bZIP

The basic leucine zipper (bZIP) transcription factor family is one of the largest and most diverse groups in higher plants and presents in all eukaryotes [447]. Many bZIP TFs have also been identified in various plant species, including rice, maize, wheat, tomato, soybean, strawberry (*Fragaria ananassa*), banana, and rapeseed [448,449,450,451,452,453,454]. In *A. thaliana*, the genome contains approximately four times more bZIP genes than other model organisms, such as *Saccharomyces cerevisiae*, *Caenorhabditis elegans*, and *Drosophila melanogaster* [447].

Plant bZIP TFs are characterized by a highly conserved domain consisting of a leucine zipper motif flanked by basic amino acids. The leucine zipper is a helical structure marked by leucine residues at every seventh position, forming a repetitive pattern [455]. Two leucine zipper domains interact to create a parallel, helical-coiled coil stabilized by hydrophobic interactions, which combines the basic regions to form a functional DNA-binding site [456,457].

Several classification methods for bZIP TFs are typically based on specific amino acid composition, DNA binding sites, conserved sequence motifs, and phylogenetic relationships. For example, in *A. thaliana*, the bZIP family is divided into ten distinct groups, A, B, C, D, E, F, G, H, I, and S, based on amino acid sequence similarity and protein structure within the bZIP domain [458]. Each subgroup of bZIP TFs performs specific functions across different plant species. For instance, bZIP TFs in group A activate the ABA signaling pathway, which regulates the expression of stress-responsive genes and significantly improves plant drought resistance [459]. *B. rapa* and rice provide additional examples of bZIP transcription factor classification. In *B. rapa*, the bZIP family has been categorized into nine distinct groups based on specific amino acid composition, including glutamine (Q), aspartic acid (D), proline (P), asparagine (N), serine (S), glycine (G)-rich domains, the presence of a transmembrane (Tm) domain, and the occurrence of low complexity regions (LCRs) or their absence [460]. In contrast, the bZIP TFs in *rice* have been classified into eleven groups (I–XI) based on their basic structural features, the amino acid sequence of the hinge region, and DNA binding site characteristics [449].

The bZIP TFs are crucial regulators in plant stress responses, helping plants withstand various environmental stresses such as drought, salinity, waterlogging, cold, and exposure to heavy metals, and play a vital role in enhancing plant resilience to these adverse conditions [461]. For instance, transgenic *A. thaliana* plants overexpressing CabZIP from pepper exhibit enhanced tolerance to *Pseudomonas syringae* Pv. *tomato* DC3000 as well as increased resistance to drought and salt stresses [398]. Similarly, in tomato, the bZIP proteins *SlAREB1* and *SlAREB2* respond to drought and salinity, with mutants overexpressing *SlAREB1* exhibiting greater tolerance to these stresses compared to wild-type plants [398]. In transgenic *A. thaliana* plants, overexpressing wheat *TabZIP60* shows significant improvements in tolerance to salt, drought, and freezing stresses, with the study revealing that *TabZIP60* binds to ABA-responsive cis-acting elements (ABREs) of ABA-responsive genes [462,463]. Moreover, the overexpression of a constitutively active form of *OsbZIP46CA1* using a multigene assembly approach significantly enhances drought tolerance in rice [464].

In soybean, four bZIP genes (*GmbZIPE1*, *GmbZIPE2*, *GmbZIP105*, and *GmbZIP62*) contribute to defense against Asian soybean rust (ASR) disease by regulating ASR-related gene expression [465]. In cassava (*Manihot esculenta*), overexpression of *MebZIP3* and *MebZIP5* confers improved resistance to cassava bacterial blight disease, while their silencing reduced defense-related gene expression, leading to a disease-sensitive phenotype [398]. Expression analysis indicated that *MebZIP3* and *MebZIP5* are induced by salicylic acid, H_2_O_2_, and *Xanthomonas axonopodis* Pv. *manihotis* [466].

In rice, three bZIP TFs that exhibit high similarity to *AtTGA2* interact with *AtNPR1* through yeast two-hybrid genetic analysis, suggesting they may trigger SA signaling in a manner similar to *A. thaliana* [467]. In tobacco, the D1 domain of the bZIP TF BZI-1 facilitates interaction with the ankyrin-repeat protein ANK1, potentially influencing defense mechanisms [468]. Transgenic *A. thaliana* plants overexpressing *CabZIP* from pepper exhibit improved tolerance to *Pseudomonas syringae pv. tomato* DC3000, as well as increased drought and salt resistance [396].

Furthermore, bZIP TFs play a critical role in ABA signaling, as demonstrated by the rapid expression of ABA-induced genes (*LEA* and *Rab16*) in rice plants overexpressing *OsbZIP42*, which also show improved drought tolerance due to ABA-dependent modifications [469]. In grape, *VvbZIP23* was identified as a key regulator of stress responses, with its expression strongly induced by drought, salinity, cold, ABA, ET, JA, and SA [470].

The extensive diversity and functional versatility of the bZIP transcription factor family present significant opportunities for advancing plant science and agriculture. Future studies focusing on the unique structural features and subgroup-specific functions of bZIP TFs across different species will provide valuable insights for developing resilient and high-yielding plant varieties.

### 3.6. MADS-Box

MADS-box TFs are one of the most prominent gene families found in plants, and they are also present across various eukaryotic organisms and some prokaryotes [471]. The MADS-box gene family is a significant group of homeotic TFs that regulates plant development. It was named after its founding members—MCM1 from *Saccharomyces cerevisiae*, AGAMOUS from *A. thaliana*, DEFICIENS from *Antirrhinum majus*, and SRF from *Homo sapiens*—with the term “MADS” introduced by Schwarz-Sommer et al. to reflect the conserved DNA-binding domain among these proteins [472].

The MADS domain has a 60-amino-acid-long N-terminus, and it recognizes the CArG-box DNA motif (CC[A/]6GG) in the target genes. MADS-box TFs are divided into two main types: Type I and Type II. Type I proteins are abundant and yet have primarily unclear functions in plants. In contrast, Type II proteins are well studied and divided into the MIKC^C^ and MIKC* subgroups [473]. MIKC-type proteins are distinguished by their complex structure, which contains four distinct domains: MADS, keratin-like (K), intervening (I), and C-terminal (C) [474,475,476]. These domains contribute to the multifunctional roles of MIKC-type genes, which are critical for various stages of plant development, from vegetative growth to reproductive processes, including responses to environmental stresses [477]. Additionally, the K-box domain, approximately 70 amino acids long and divided into three subtypes (K1, K2, and K3), is often used to differentiate between Type I and Type II MADS-box genes [478].

According to the ABCDE model of flower development, MADS-box genes play a pivotal role in reproductive processes, including female gametophyte development, embryogenesis, and endosperm formation. Type II MADS-box genes are essential in floral organ identity, meristem differentiation, and fruit development [479]. Functional analyses have revealed that various MIKC genes regulate flowering time in crops. Phylogenetic studies have underscored the diversity and evolutionary significance of these genes by highlighting their crucial functions throughout the plant life cycle, from embryogenesis to gametophyte development and fruit ripening [480,481,482].

MADS-box TFs are critical regulators of plant responses to various abiotic stresses. In *A. thaliana*, the gene *AGL16* is predominantly expressed in guard cells; however, it is downregulated during drought stress. It binds to the CArG-box in the CYP707A3, AAO3, and SDD1 promoters to regulate stomatal density and ABA levels [483]. Additionally, *AGL16* acts as a negative regulator of salt stress by suppressing HSPs and ABA signaling pathways through the downregulation of salt stress-related genes in *A. thaliana* [484].

In rice, *OsMADS25* enhances tolerance to salt and oxidative stresses, while *OsMADS23* acts as a positive regulator in the osmotic stress response. *OsMADS23* interacts with the SnRK2-type protein kinase SAPK9, which phosphorylates it, increasing its stability and transcriptional activity. Activated through the ABA signaling cascade, *OsMADS23* promotes the expression of ABA and proline biosynthesis-related genes, including *OsNCED2* and *OsP5CR* [485]. Similarly, *OsMADS57* interacts with *OsTB1* to regulate the defense gene *OsWRKY94* and the organogenesis gene *D14* during cold stress, enhancing cold tolerance while maintaining a balance under normal conditions [486]. In citrus, the MADS-box TF *PtrANR1* improves drought tolerance by increasing IAA levels and promoting root growth by interacting with the CArG-box in the *PtrAUX1* promoter [487]. In sheepgrass (*Festuca ovina*), MADS-box TFs contribute to drought stress adaptation by enhancing root growth and drought tolerance [488].

As functioning in biotic stresses, silencing *NbMADS1* reduces the tobacco plant resistance to *Phytophthora nicotianae*, indicating that *NbMADS1* is a positive regulator of systemic resistance [489]. In rice, plants with reduced expression of *OsMADS26* exhibit enhanced resistance to *Magnaporthe oryzae* and *Xanthomonas oryzae*, while overexpression of *OsMADS26* moderately increases susceptibility to *M. oryzae* [490]. These findings confirm the role of genes in negatively controlling disease resistance. Moreover, MADS-box genes show significant expression changes in response to pathogen infections in bread wheat. For example, *TaMADS19* expression increases 7- to 10-fold following infection by *Septoria tritici*, suggesting its involvement in pathogen response. Conversely, *TaMADS117* is downregulated 3- to 7-fold during powdery mildew infection, indicating a distinct regulatory role [491].

The MADS-box transcription factor family offers a profound opportunity to enhance our understanding of plant development and adaptation. By leveraging their pivotal roles in reproductive processes, floral organ identity, and stress responses, these TFs can be harnessed to optimize flowering time, improve crop yields, and adapt plants to challenging environments.

### 3.7. MYB Family

MYB (v-Myb myeloblastosis viral oncogene homolog) TFs are considered one of the largest TFs in plants [492]. MYB proteins are characterized by a conserved DNA-binding domain composed of 1–4 MYB repeats (R1–R3), and these proteins are ubiquitous in eukaryotes, and an exceptionally high number of their homologs are found in plants, forming one of the most prominent transcription factor superfamilies [493,494,495,496]. The MYB transcription factor family is crucial in growth, development, and metabolic regulation in plants [497,498].

The MYB superfamily of TFs is generally divided into four major families based on the number of MYB repeats they contain: 2R-MYB (R2R3-MYB), 3R-MYB (R1R2R3-MYB), 4R-MYB (R1R2R2R1/2-MYB), and MYB-related (MYBR/1R-MYB) [495]. Among these, the MYBR and 2R-MYB proteins constitute the largest families, typically comprising dozens to hundreds of members in higher plants. In contrast, the 3R-MYB and 4R-MYB families are much smaller, with only a few members in some species [496]. The 2R-MYB family, initially discovered in maize, where it was linked to anthocyanin biosynthesis, has since been identified across a broad range of plant species, representing one of the largest transcription factor families in plant genomes [493]. Despite the extensive study of 2R-MYB proteins, the functions of 4R-MYB genes remain largely unknown [499]. Among the MYB proteins, the R2R3-MYB TFs are the most abundant in plants. Their DNA-binding region comprises two homologous MYB domains, R2 and R3, which work synergistically to bind target DNA sequences [500,501]. Most R2R3-MYB proteins also possess transcriptional activation domains at their C-terminus, critical for regulating plant cell differentiation, secondary metabolism, hormone responses, and resistance to various biotic and abiotic stresses [495].

MYB TFs are involved in plant development and defense responses, including the cell cycle, cell morphogenesis, central circadian oscillation, and the regulation of stress signaling [502,503]. Moreover, it has a pivotal role in absorbing nutrient substances, regulating salinity, drought resistance, trichome growth, stamen development, leaf senescence, and the biosynthesis of flavonoids [504]. Research has shown that MYB TFs play a significant role in enhancing stress tolerance by regulating the biosynthesis of cuticle and suberin in plants [505,506,507]. Recent studies have identified 2120 R2R3-MYB genes across six *Brassica* species (including 130 in *A. thaliana*, 425 in *B. napus*, 247 in *B. oleracea*, 236 in *B. rapa*, 248 in *B. nigra*, 412 in *B. carinata* and 422 in *B. juncea*). However, the number of these genes in *A. thaliana*, *B. rapa*, and *B. napus* differs from previous reports [508,509]. In *B. rapa* ssp., functional analysis revealed that the MYB transcription factor *BrTDF1* is involved in tapetum development, a critical process for anther and pollen formation, as tapetum is a vital nutrient provider during these stages [510].

MYB TFs play vital roles in plant responses to abiotic and biotic stresses. In *A. thaliana*, stress-induced flavonoid accumulation serves as a positive drought response [511]. Overexpression of *PAP1* or *MYB12*, key regulators of flavonol synthesis, reduces water loss, improving drought tolerance [512]. In apple, *MdoMYB121* is upregulated during drought stress, and its overexpression in tomato and apple enhances drought resistance [513]. Similarly, *MdSlMYB1* is induced by drought and other abiotic stresses, promoting tolerance by activating stress- and auxin-responsive genes [514]. In rice, MYB TFs such as *OsMYB4*, *OsMYB3R-2*, and *OsMYB2* are upregulated during drought, and their overexpression improves drought resistance [515,516]. Additionally, *OsMYB48-1* is induced by ABA, H_2_O_2_, dehydration, and polyethylene glycol (PEG), with minimal expression under salt and cold stress [517]. Transgenic rice overexpressing *OsMYB48-1* exhibits enhanced tolerance to drought and salinity [517]. *OsMYB55* overexpression in maize improves drought and heat tolerance by upregulating stress-related genes, and *OsMYB6* overexpression in rice increases salt and drought resistance [518,519].

In sorghum (*Sorghum bicolor*), MYB TFs contribute to responses against abiotic and biotic stresses. *SbMYB60* overexpression activates monolignol biosynthesis in drought-tolerant sorghum [520]. Additionally, sorghum produces 3-deoxyanthocyanidin phytoalexins in response to fungal pathogens such as *Colletotrichum sublineolum*, a process requiring the MYB TF *Yellow Seed 1 (Y1)*. Transgenic maize expressing the *Y1* gene produces 3-deoxyanthocyanidins, enhancing resistance to leaf blight [521].

In wheat, overexpression of the R2R3-MYB TF *TaRIM1* improves resistance to *Rhizoctonia cerealis* infection [522]. Similarly, in response to various pathogens, including *Pseudomonas syringae pv. tomato*, *Hyaloperonospora parasitica*, and *Plectosphaerella cucumerina*, plants exhibit increased expression of MYB72, along with callose formation and activation of JA-mediated defense pathways [523].

In rice, OsDRxoc1 enhances resistance to *Xanthomonas oryzae pv. oryzicola* (Xoc) while suppressing resistance to *X. oryzae pv. oryzae* (Xoo) [524], whereas *AtMYB44* in *A. thaliana* increases tolerance to *Myzus persicae;* however, it reduces resistance to *Erwinia carotovora* [525,526]. *AtMYB30* is induced by ROS and regulates immune responses by targeting lipid transfer proteins (*LTPG1* and *LTPG2*) and transfer proteins [527].

Additionally, *AtMYB15*, *AtMYB30*, and *AtMYB73* in *A. thaliana*, along with *MdMYB30* in apple and *PacMYBA* in cherry (*Prunus avium*), enhance resistance to *Pseudomonas syringae pv. tomato* (Pst) DC3000 in transgenic *A. thaliana* [528,529]. In cotton, *GhMYB4*, *GhMYB36*, and *GhODO1* improve resistance to *Verticillium dahliae* by modulating multiple defense mechanisms [530,531,532]. Furthermore, *AtMYB13* is induced by *Pst* DC3000 as well as JA and SA signaling, yet remains upregulated even in JA/SA-insensitive mutants, suggesting its role in a JA/SA-independent defense pathway [524]. Meanwhile, *GhMYB33* suppresses anthocyanin biosynthesis genes, while *GhMYB43* negatively regulates JA signaling and lignin biosynthesis genes, both contributing to *V. dahliae* resistance [533].

The MYB transcription factor family represents a promising target for advancing plant resilience and productivity. Their diverse roles in stress tolerance, secondary metabolism, and developmental processes offer the potential for improving crop adaptation to environmental challenges. Future exploration of the less-studied MYB subfamilies and their regulatory mechanisms will provide valuable insights for developing stress-tolerant and high-yielding crops tailored for sustainable agriculture.

**Table 1:** Transcription factors involved in plant stress responses and development across different species.

TF	Plant	Gene/Genotype/TF	Function	References
NAC	*A. thaliana*	*ATAF1* *overexpression*	Decrease resistance while loss-of-function mutants increase resistance to *B. cinerea*, *Pst* DC3000, and *Alternaria brassicicola*	[534]
		*ATAF2* *overexpression*	Decrease resistance to *Fusarium oxysporum*	[535]
		ANAC062/NTL6 overexpression	Increased resistance to *Pst* DC3000	[536]
		*ANAC055* overexpression	Drought, high salinity, ABA signaling	[537]
		CBNAC/NTL9	Stomatal immunity to *Pst* DC3000	[538,539]
		*GmNAC20* overexpression	Salt and freezing tolerance	[540]
		NAC4 overexpression	Increase *Pst* DC3000-induced cell death	[541]
		*RD26* overexpression	Drought, salt, ABA signaling	[542]
	Rice	*OsNAC4* overexpression	Drought, salt, cold tolerance	[543]
		*OsNAC6* overexpression	Drought and salt tolerance	[544]
		*ONAC10* overexpression	Drought, high salinity, low-temperature tolerance	[545]
		*SNAC2* overexpression	Salt, drought, salinity, cold, and wounding	[546]
	Tomato	SlSRN1	Silencing attenuated resistance to *B. cinerea* and *Pst* DC3000	[547]
		JA2	JA2-suppressed plants increased susceptibility to *Pst* DC3118	[548]
	Wheat	TaNAC21/TaNAC22	Silencing increased resistance to *Puccinia striiformis* f. sp. *tritici*	[549,550]
		TaNAC6s overexpression	Enhance resistance to *Blumeria graminis* f. sp. *tritici*	[300]
HSF	*A. thaliana*	Hsf30 (TF)	Help to maintain protein stability and cellular function under high temperature	[551]
		Hsp70 (HSP)	Prevent protein aggregation, particularly under stress conditions like heat, drought, and salinity	[552]
		*PtHSP17.8* *overexpression*	Increases survival rate and root length under heat and salt stresses	[553]
		*MsHSP16.9* *overexpression*	Improves the tolerance of a plant to heat by alleviating the damages of ROS and regulating the expression levels of stress-related genes	[554]
	Tobacco	*RcHSP17.8* *overexpression*	Resistant to high temperatures and osmotic stresses	[555]
	Tomato	*AtHsfA1b* *overexpression*	Improve resistance to chilling in transgenic tomato	[556]
	Rice	*HsfA4a* *overexpression*	Enhance Cd tolerance	[544]
	Wheat			
WRKY	*A. thaliana*	*AtWRKY57*	Enhance drought tolerance	[557]
		*AtbHLH17*	Upregulate under drought and oxidative stress	[558]
		*AtWRKY28*		
		*AtWRKY23*	Participate in root development by controlling auxin distribution	[559]
		*VvWRKY11* *overexpression*	Higher tolerance to water stress induced by mannitol and response to dehydration stress	[560]
	Rice	*OsWRKY45* *overexpression*	Enhance salt and drought tolerance and increase disease resistance	[385]
		*OsWRKY53*	Involved in pathogen defense pathways	[385]
		*WRKY72* *overexpression*	Increase sensitivity to sugar starvation stress	[561]
		*OsWRKY11* *overexpression*	Decrease plant height, tolerance to drought stress	[562]
	Soybean	*GmWRKY20*	Induced by ABA, salt, cold, and drought, and involved in several abiotic stress-related responses	[385]
		*GmWRKY13*	Sensitive to salt and mannitol, negative regulator in ABA signaling	[563]
		*GmWRKY54*	Tolerance to salt and drought	[563]
	*B. campestris*	*BcWRKY46*	Tolerance to salt and drought	[564]
	*Zea mays*	*ZmWRKY17*	Reduce salt tolerance	[565]
	*Malus domestica*	*MdWRKY30*	Tolerance to salt and osmotic stress	[566]
		*MdWRKY100*	Sensitive to salt	[567]
Ap2/ERF	*A. thaliana*	*AtERF7* *overexpression*	Reduce the sensitivity of defense cells to ABA and increase water loss	[568]
		*AtERF9* *AtPDF1.2*	Enhance resistance to *Botrytis cinerea*	[439]
		*AtC4H* *At4CL1*	Key players in phenylpropanoid metabolism and cell wall formation	[569]
		*ZmEREB20*	Enhance ABA sensitivity and cause delayed seed germination under salt stress by regulating abscisic acid and gibberellin-related genes	[441]
	Rice	*OsERF71* *overexpression*	Reduce water loss, resulting in increased tolerance to drought stress	[570]
		*OsDREB2B* *overexpression*	Improve drought tolerance	[571]
		*OsERF19* *overexpression*	Increase the tolerance of plants to salt stress	[572]
		*OsDREB2A* *OsDREB1F*	Water scarcity and high salt stress tolerance	[573]
	Soybean	*GmERF113*	Enhance resistance to infection of *Phytophthora sojae*	[437]
	Apple	*MdERF11*	Positively regulate defense responses against *Botryosphaeria dothidea* by promoting SA biosynthesis	[440]
bZIP	*A. thaliana*	*AtbZIP15* *AtbZIP35* *AtbZIP36* *AtbZIP37* *AtbZIP38*	Abscisic acid biosynthesis and stress signaling	[574,575]
		*AtbZIP17* *AtbZIP28*	Regulate the unfolded protein response pathway	[576,577]
		*AtbZIP10*	Pathogen defense	[578]
	Barley	*HvbZIP56*	Regulate zinc deficiency	[579]
		*HvbZIP62*		
	Rice	*OsbZIP23* *overexpression*	Improve drought resistance and salt tolerance	[580]
		*OsbZIP46*	Mediate in ABA signaling and drought resistance	[581]
		*OsbZIP72* *overexpression*	ABA sensitivity and drought resistance	[581]
	Soybean	*GmbZIP14*	Salt, drought, and low-temperature stress responses	[582]
		*GmbZIP146*	Flowering, salt and drought stress responses	[583,584,585]
		*GmbZIP53*	ABA signaling, salt, and low-temperature stress responses	[586]
		*GmbZIP45*	Pathogen response	[587]
MADS-box	*A. thaliana*	*CmANR1* *overexpression*	Lateral root formation and growth, auxin biosynthesis, auxin polar transport, calcium ion signaling, ethylene biosynthesis, and cell cycle-related processes	[588]
		*AtAGL11*	Regulate fruit and transmitting tract development	[589]
	*B. napus*	*BnaAGL11*	Cause smaller, curly leaves and accelerated leaf senescence	[590]
		*Bna.C09.AGL11* *overexpression*		
		*Bna.A04.ABI5* *Bna.A05.ABI5*, *Bna.C04.ABI5-1*	Leaf senescence	[590]
	Rice	*OsMADS14*, *OsMADS15,*	Floral meristem identity	[591]
		*OsMADS18*	Germination, tillering, and inflorescence architecture	[592]
	Sweet potato	*IbMADS1*	Early-developing tuberous roots	[593]
		SRD1	Metaxylem and cambium cell formation	[594]
MYB family	*A. thaliana*	*AtMYB96*	Drought resistance by modulating cuticular wax biosynthesis	[506,507,595]
		*AtMYB41* *overexpression*	Synthesis and deposition of suberin-type aliphatic monomers in leaves rather than the usual cutin-type, which may result in discontinuities in the leaf cuticle and increase desiccation sensitivity in transgenic plants	[596,597]
		*AtMYB16 AtMYB106*	Cuticle biosynthesis	[505]
		*AtMYB2* *AtMYB20* *AtMYB73* *AtMYB74*	Regulate salt tolerance by modulating gene expression under salt stress	[598]
	Rice	*OsMYB2* *overexpression*	Enhance cold resistance in transgenic rice	[599]
		*OsMYB102*	Represses the ABA	
	Apple	*MdMYB108L*	Improve cold tolerance	[600]
	Tomato	*SlMYB49*	Manage stress responses	[601]
	Pear	*PbrMYB169*	Enhance lignification in the cell matrix of fruit	[602]

## 4. Other Molecules Participate in Signaling and Stress Response

Apart from phytohormones and TFs, numerous other molecules contribute to plant signaling, growth, development, and stress responses. Hydrogen peroxide interacts with thiol-containing proteins, activating signaling pathways and transcription factors that influence gene expression and cell cycle regulation [603]. During pathogen attacks, a temporary increase in H_2_O_2_/reactive oxygen species (ROS) levels triggers a localized defense response, partly through NADPH oxidase activation. Elevated H_2_O_2_/ROS levels also contribute to the hypersensitive response (HR), leading to programmed cell death (PCD) at infection sites [604]. Beyond stress signaling, H_2_O_2_/ROS regulates plant growth and development, including morphogenesis and cellulose-rich cell wall differentiation. Extracellular Cu/Zn superoxide dismutase (SOD) helps maintain H_2_O_2_ balance, thereby influencing plant development [605].

Polyamines are small, positively charged molecules present in all domains of life, including plants. Transcriptomic studies in *A. thaliana*, wheat, and tomato reveal that polyamine metabolism genes are differentially expressed under stress conditions, indicating their role in abiotic stress responses [606,607,608]. Certain stress-related transcription factors regulate ADC genes in different species. For instance, an NAC family member binds the ADC promoter in *Poncirus trifoliata*, HEAT-SHOCK FACTOR A3 (HSFA3) regulates Putrescine (Put) biosynthesis in rice, and ZAT12 mediates cold-induced ADC expression in *A. thaliana* [609,610]. Polyamines also play a complex role in plant–microbe interactions, with both plants and pathogens synthesizing these compounds. Pathogen-induced Put accumulation is primarily driven by ADC gene upregulation. Supporting its importance in plant defense, *A. thaliana* plants with silenced ADC genes exhibit increased susceptibility to *Botrytis cinerea* [611].

Beyond being a nutrient, nitrate functions as a signaling molecule that regulates plant growth and development [612]. The *A. thaliana* dual-affinity nitrate transporter NRT1.1 (CHL1/NPF6.3) acts as a nitrate sensor, activating nitrate assimilation and transporter genes to optimize nutrient uptake [613]. NRT1.1 also influences auxin transport, with nitrate inhibiting auxin movement in a dose-dependent manner, integrating nitrate and auxin signaling. Therefore, NRT1.1 functions as a key regulator that coordinates nitrate signaling and transport with auxin signaling and transport, ultimately controlling plant growth and development [614]. The expression and activity of NRT1.1 are modulated by TFs, kinases, and phosphatases under abiotic stress. For instance, the TF STOP1 enhances NRT1.1 transcription under low rhizosphere pH, increasing nitrate uptake [615]. During drought stress, CPK6 phosphorylates NRT1.1 at Thr447, reducing its transport activity, while SnRK2 kinases phosphorylate NRT1.1 at Ser585 in response to ABA signaling, impairing nitrate transport during stress adaptation [616].

Nitric oxide (NO) also plays a crucial role in balancing plant development and stress responses through post-translational modifications (PTMs) such as S-nitrosation and Tyr-nitration [617,618]. NO interacts with hormones like ABA in stress signaling, activating transcription factors that regulate stress-responsive genes. For example, NO-induced activation of *AtAO3* enhances drought tolerance in *A. thaliana* [619]. Exogenous NO application has been shown to improve resilience against flooding, heavy metal toxicity, and mineral deficiencies [620]. NO is also involved in plant–pathogen interactions, modulating metabolic functions during pathogen attacks. In tobacco plants infected with *Pseudomonas syringae*, NO and nitrite influence primary metabolism during the hypersensitive response, highlighting the role of NO in immune signaling [621].

Glutathione (GSH), often called the “master antioxidant” or “super defender”, is a widely occurring non-protein thiol tripeptide present in both prokaryotic and eukaryotic organisms [622,623]. In response to SA treatment, pea seedlings exhibit increased GSH levels, decreased oxidized glutathione (GSSG), and an elevated GSH:GSSG ratio [624]. Similarly, SAR induced by iso-nicotinic acid (INA), an SA analog, or infection with *Pseudomonas syringae* leads to higher total glutathione content and an increased GSH:GSSG ratio [625]. Ethylene also influences GSH biosynthesis. Exposure to ethylene increases the production of GSH, while ethylene signaling mutants (ein2-1) show reduced expression of γ-glutamyl synthetase (γ-ECS), resulting in lower GSH levels [626]. In transgenic tobacco plants with elevated GSH content, PR4 (an ethylene signaling marker) and ACC oxidase (an enzyme converting ACC to ET) are upregulated, demonstrating a synergistic interaction between GSH and ET [627].

Some sugar molecules also contribute to plant signaling and stress responses. Sucrose plays a regulatory role in metabolism and development, primarily through SnRK1 and possibly the target of rapamycin (TOR) pathway [628]. SnRK1 integrates stress and energy signals, leading to transcriptional reprogramming and growth control [629]. Trehalose, a non-reducing disaccharide, serves as a signaling metabolite in plant interactions with pathogens, symbiotic microorganisms, and herbivorous insects [630]. Additionally, it plays a role in plant responses to cold and salinity stress while also influencing stomatal regulation and water-use efficiency. Drought-resistant maize varieties exhibit faster TPS and TPP gene expression induction than drought-sensitive cultivars, highlighting the role of metabolism in drought resilience [631].

## 5. Future Prospects

Plants that thrive in extreme environments, such as extremophiles and desiccation-resistant species, have evolved unique genetic adaptations to withstand harsh conditions. Introducing these genes into agriculturally important crops like wheat, rice, and maize can significantly enhance their stress tolerance.

For instance, wheat plants overexpressing the *HVA1* gene have demonstrated increased tolerance to both drought and heat stress. This tolerance is associated with the upregulation of transcription factors, such as DREB under drought stress and HsfA6 under heat stress [632]. Notably, the overexpression lines exhibited ABA sensitivity, suggesting that HsfA6 contributes to heat tolerance via the ABA-mediated pathway. These findings highlight the potential role of HVA1 in heat stress signaling, offering a promising approach for engineering multi-stress tolerance in crop plants [632].

Similarly, the transcription factor XvSAP1, derived from *Xerophyta viscosa*, enhances drought tolerance in transgenic sweet potato without causing adverse phenotypic or yield-related effects. These transgenic lines serve as valuable resources for cultivating drought-tolerant crops in arid regions, with potential applications for other staple crops [633].

Moreover, exploring the unique functions of phytohormones and transcription factors from wild plants can provide novel strategies to improve crop resilience against both biotic and abiotic stresses. These genetic applications hold great promise for developing climate-resilient crops, improving water-use efficiency, and enhancing stress adaptation mechanisms in agriculture, ultimately contributing to sustainable food production in increasingly challenging environments.

## 6. Conclusions

Plant phytohormones and TFs are essential in plant development and regulating responses to various abiotic and biotic stresses. These molecules coordinate complex networks that help plants adapt to challenging environmental conditions by modulating gene expression and activating defense mechanisms. Understanding the intricate interactions between phytohormones and TFs is crucial for developing strategies to enhance crop stress tolerance. With advancements in biotechnology, such as gene editing and omics approaches, there is significant potential to engineer plants that are more resilient to climate change and other stressors, ultimately improving agricultural productivity and sustainability.

## Data Availability

Data are contained within the article.
